# Liquid–Liquid Dispersion Performance Prediction and Uncertainty Quantification Using Recurrent Neural Networks

**DOI:** 10.1021/acs.iecr.4c00014

**Published:** 2024-04-22

**Authors:** Fuyue Liang, Juan P. Valdes, Sibo Cheng, Lyes Kahouadji, Seungwon Shin, Jalel Chergui, Damir Juric, Rossella Arcucci, Omar K. Matar

**Affiliations:** † Department of Chemical Engineering, Imperial College London, London SW7 2AZ, U.K.; ‡ CEREA, École des Ponts ParisTech-EdF R&D, Champs-sur-Marne 77455, France; § Department of Mechanical and System Design Engineering, Hongik University, Seoul 04066, Republic of Korea; ∥ Centre National de la Recherche Scientifique (CNRS), Laboratoire Interdisciplinaire des Sciences du Numérique (LISN), Université Paris Saclay, Orsay 91400, France; ⊥ Department of Applied Mathematics and Theoretical Physics, University of Cambridge, Cambridge CB3 0WA, U.K.; # Department of Earth Science & Engineering, Imperial College London, London SW7 2AZ, U.K.

## Abstract

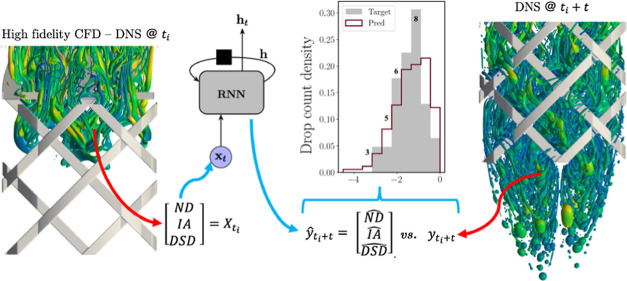

We demonstrate the application of a recurrent neural network (RNN) to perform multistep and multivariate time-series performance predictions for stirred and static mixers as exemplars of complex multiphase systems. We employ two network architectures in this study, fitted with either long short-term memory and gated recurrent unit cells, which are trained on high-fidelity, three-dimensional, computational fluid dynamics simulations of the mixer performance, in the presence and absence of surfactants, in terms of drop size distributions and interfacial areas as a function of system parameters; these include physicochemical properties, mixer geometry, and operating conditions. Our results demonstrate that while it is possible to train RNNs with a single fully connected layer more efficiently than with an encoder–decoder structure, the latter is shown to be more capable of learning long-term dynamics underlying dispersion metrics. Details of the methodology are presented, which include data preprocessing, RNN model exploration, and methods for model performance visualization; an ensemble-based procedure is also introduced to provide a measure of the model uncertainty. The workflow is designed to be generic and can be deployed to make predictions in other industrial applications with similar time-series data.

## Introduction

Multiphase dispersion processes, and in particular liquid–liquid (L–L) mixing, are of central importance to a broad range of industrial applications, ranging from microscopically manufactured (“structured”) emulsions in the manufacturing of fast-moving consumer goods and pharmaceuticals, to chemical reactions (e.g., nitration, sulfonation, etc.) in the energy sector.^1,2^ These operations greatly depend on several key performance indicators such as the interfacial area of the dispersed phase governing mass transfer-controlled reaction rates, as well as the droplet size distribution (DSD) and count determining the stability and physical properties of emulsions. Consequently, numerous studies have focused on developing predictive (semi)-empirical correlations to estimate metrics such as the mean/maximum drop size based on a given set of flow conditions and fluid properties^3−6^ and a broad range of mixing devices, designs, and flow regimes.^7−11^ Lately, robust numerical frameworks have been developed aiming to improve the predictive capabilities of previous empirical models by providing a physics-based understanding of the governing phenomena via computational fluid dynamics (CFD) simulations and population-balance modeling.^12−14^

While it is true that substantial ground has already been covered, both experimentally and numerically, multiple challenges still exist when modeling complex and industrially relevant mixing processes. A prevalent scenario in L–L systems is the presence of surface-active agents (surfactants), originating either as contaminants or additives. Such systems require a much more comprehensive computational framework to accurately describe the dispersion dynamics unfolding, such as the inclusion of equations of state and surfactant mass transport modeling, to account for the intertwined effect between interfacial tension and surfactant concentration. Analogous nonidealities in multiphase mixing flows, such as highly concentrated, turbulent or non-Newtonian systems, lead to a similar challenge. Consequently, this situation gives rise to a challenging trade-off between model robustness and accuracy on the one hand and resource consumption on the other. Therefore, it is unsurprising that no general model has been established thus far to provide sufficiently accurate predictions of the dispersion performance under analogous scenarios.

Fortunately, the rapid development of artificial intelligence (AI) and data-driven techniques has granted us access to a powerful and cost-effective toolkit of computational alternatives to circumvent the challenges set out above. In recent years, with the increase in available simulation data, machine learning (ML) models have become popular tools to accelerate CFD simulations in multiple engineering fields such as chemical engineering.^15^ Among the different types of ML-based techniques, recurrent neural networks (RNNs) stand out due to their capability to handle sequential data for time-series predictions. Apart from early single/multilayer networks, also known as traditional or “vanilla” RNNs, several more variants stand out, including simpler or learner versions such as Elman and Jordan RNNs, where outputs from only one layer are fed back, as well as more complex structures such as gated recurrent unit (GRU) and long–short-term memory (LSTM) networks, which implement feature gates to improve the handling of long-term dependencies.^16^ Other RNN variations have been tailored for specific applications, thus making them less applicable for chemical engineering processes, such as Hopfield networks and NARX-type RNNs for identification and control of nonlinear dynamic systems, and adaptive networks like ABAM and transversal/recursive filters for signal processing and communications.^17^

Diving into one of the most popular variants of RNNs, LSTM networks, designed to solve the so-called vanishing problem^18,19^ seen in the “vanilla” RNNs, have been commonly used in a wide range of applications. For instance, LSTM networks have been trained using simulation data to predict scenarios including material leakage position^20,21^ and bio-oil yield of a fluidized bed.^22^ Moreover, the LSTM unit architecture has been coupled with reduced ordered modeling to model the key features underlying turbulent flows.^23^ Similarly, a combination of the two frameworks has been applied to carry out transonic aeroelastic analysis.^24^ More recently, novel models based on LSTM have been proposed to forecast the hydrodynamics of submarine prototypes^25^ and the behavior of ocean waves.^26^ Similarly, its younger “sibling”, GRU,^27^ has also risen in popularity due to their similar performance at a seemingly lower model complexity, as we will explore in detail in the following section.

Inspired by the work reviewed before, this study seeks to develop an inexpensive time-series model using RNNs by capitalizing on a comprehensive set of high-fidelity three-dimensional CFD simulations, some of which have been exploited in recent works^28−31^ to unravel the fundamental governing mechanisms underlying extensively utilized mixing systems handling L–L dispersions across a range of industrially relevant scenarios. These simulations have been conducted with a state-of-the-art DNS code, which comprises a hybrid front-tracking/level-set interface-tracking algorithm, embedded along a well-validated multiphase solver for surfactant transport at the interface and in the bulk phase.^32^ This framework unlocks an unprecedented level of detail on the interfacial dynamics unfolding and thus provides an accurate, physics-based estimation of the temporal evolution of key performance metrics, such as interfacial area growth, drop generation, and DSD. The works by Liang et al. (2022 and 2023) explored a pitch-blade stirred vessel mixer with varying impeller speeds and different surfactant profiles, whereas those by Valdes et al. (2023a and 2023b) ran simulations with an SMX static mixer considering different inlet configurations and types of surfactants.

The time-series model implemented in this work aims to supersede the high-fidelity CFD framework used in these previous works and predict the key dispersion metrics previously mentioned based solely on their initial behavior during the early stages of the process. More importantly, we intend to investigate the model’s capability to identify different initial performance signatures, inherently linked to mixer design, operational conditions, or surfactant physicochemical profile, and extrapolate their future behavior accordingly. To achieve this, we develop a general predictive workflow around three mixing performance metrics, with the implementation of RNNs at its core, as shown in Figure 3. In particular, we center our attention on LSTM units but also compare against the recently introduced GRU.^33^ The trained network is initialized with early stage high-fidelity CFD data and set to self-iterate on its output in a “rollout” procedure to generate future performance predictions. Finally, we perform an uncertainty quantification analysis on the trained model via ensemble perturbation to track the evolving model uncertainty and its performance through the prediction propagation via “rollout”. There have been studies suggesting that ensemble-based methods are an important pillar to quantify the uncertainty of models.^34−37^ However, we propose a novel method herein which is applicable to cope with the fact that the trained model iterates over its output numerous times during the prediction, and relevant details are presented in the Methodology section.

**Figure 1:**
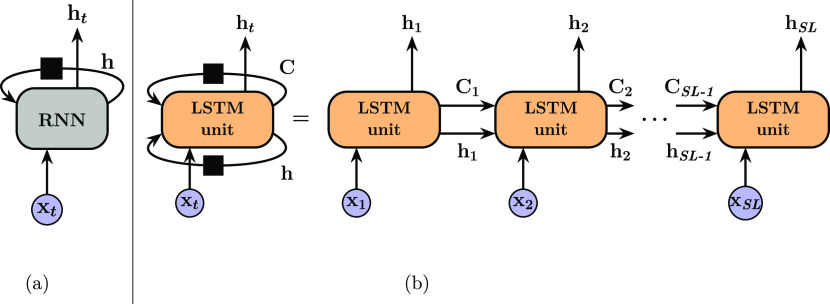
(a) Circuit diagram of an RNN with no output layers; (b) circuit diagram (left) and its unrolled view (right) until a final time-step labeled as sequence length (SL) of an LSTM network.

**Figure 2:**
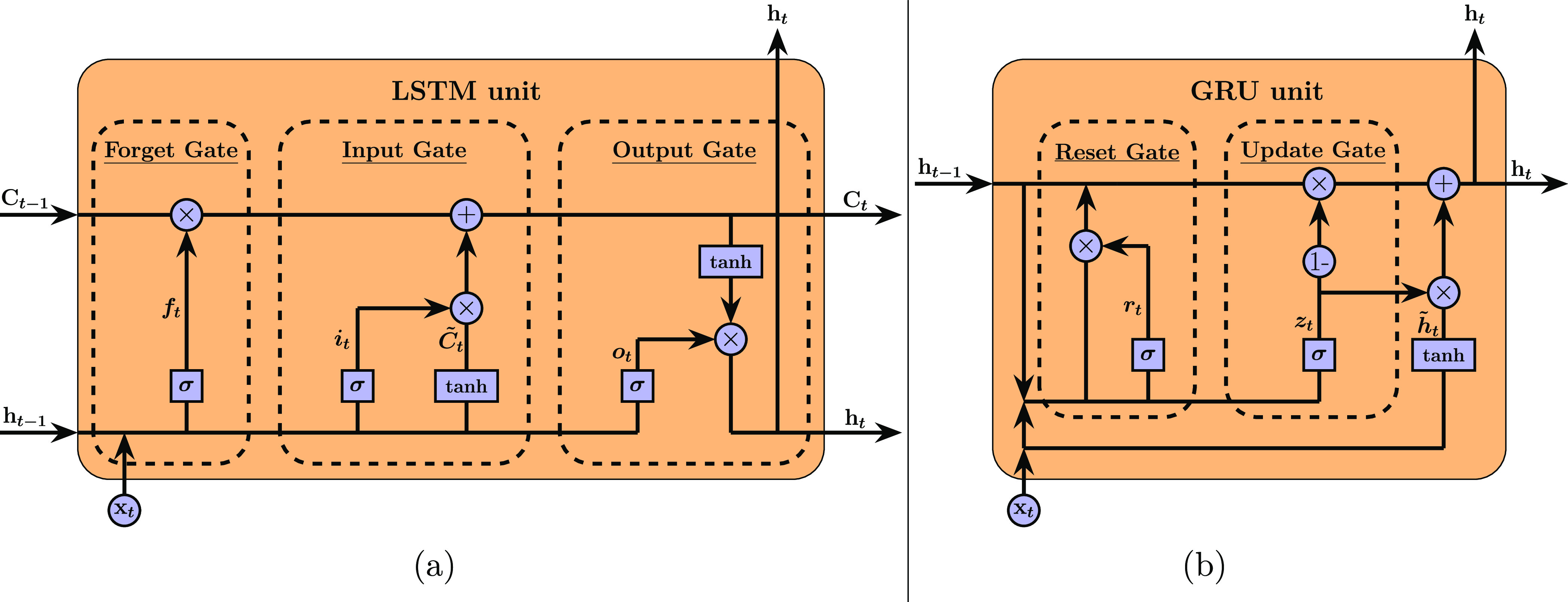
Diagram of different gates in (a) *standard* LSTM unit and (b) GRU unit.

**Figure 3:**
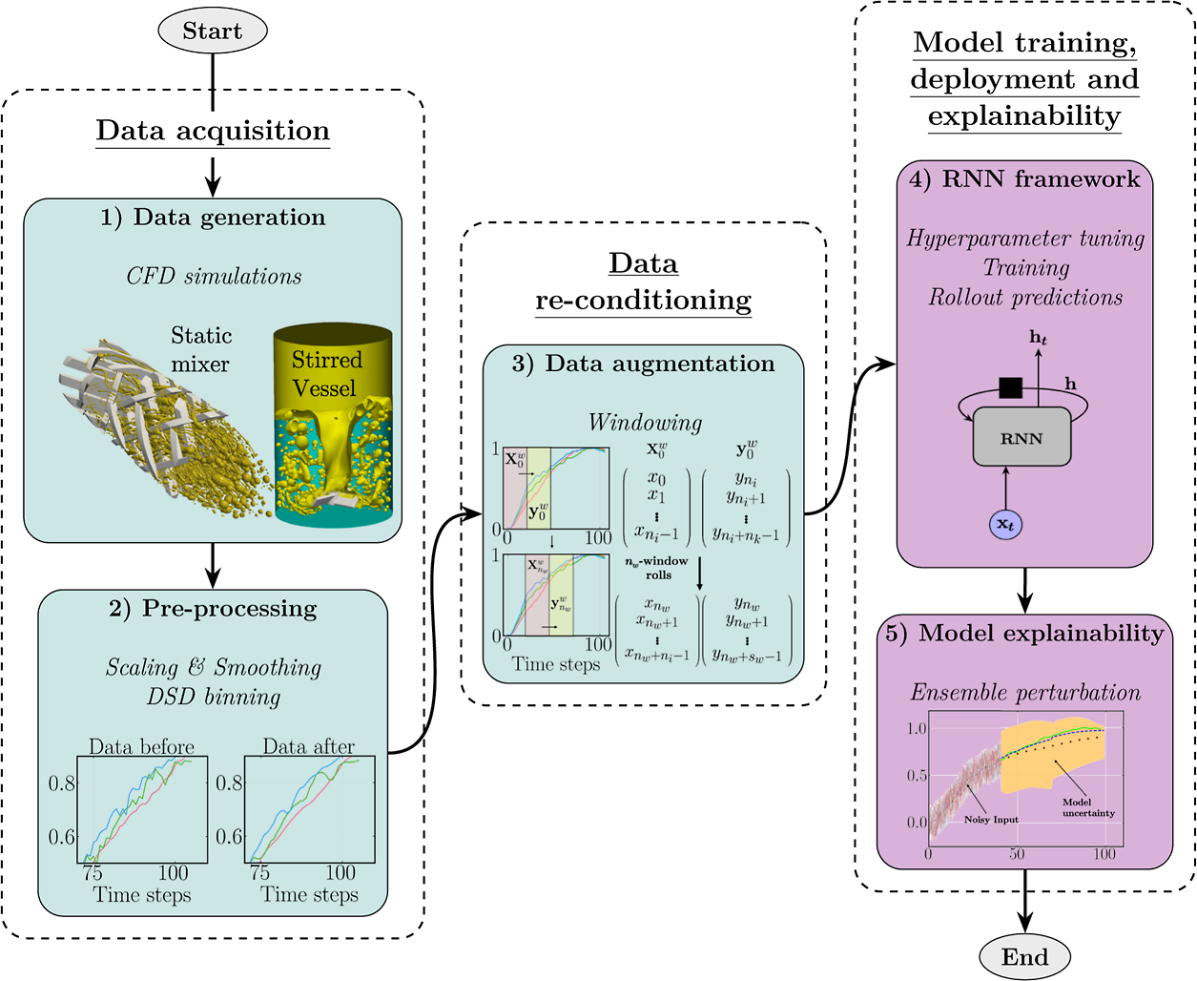
Flowchart detailing the overall framework architecture developed to train and deploy a multivariate multistep RNN model with two-phase mixing performance data from high fidelity CFD simulations. Color-coded blocks refer to general data preprocessing (teal) and model operations and exploration (violet).

The rest of this paper is organized as follows: Section 2 covers the main theoretical concepts of RNNs (focusing on LSTM and GRU units) relevant to our work; Section 3 presents the overall framework development and deployment, from CFD data acquisition, preprocessing, and reconditioning, to model training, tuning, and sequence generation via rollout methodology, discussing two separate model architectures and exploring their accuracy and uncertainty. The predictions vs CFD, which are treated as the ground truth data generated for both stirred vessels and static mixers and the corresponding discussion around the model’s explainability, are presented in Section 4. Finally, concluding remarks are given in section 5.

## Theoretical Background on RNNs: LSTM and GRU Units

We aim to perform sequence prediction via deep learning, which is different from the other types of learning problems since it imposes an order on the observations that must be preserved for model training and deployment. RNNs are specifically designed to address such problems where they have loops that allow the information from one-time-step to be passed to the next. As shown in Figure 1a, when an RNN is trained to predict a dynamical quantity in the future from the past, the network incorporates the information from the input observations **x** into the hidden state **h**, which is passed forward through time. The circuit depicts that the current state is fed back into the network influencing its future state, and the black square indicates that such an interaction takes place with a delay of one-time-step. Once the whole historical sequence, **x**_*t*_, is scanned, the RNN learns a summary, **h**_*t*_, of the relevant aspects in the past up to time *t*. Following this, an extra architecture layer, known as *output layer*, will be added to read information out of the **h**_*t*_ to make predictions.

A traditional RNN has only one hidden state, **h**_*t*_, which is sensitive to short-term inputs. However, when the sequence length grows, it becomes unable to learn the summary that connects the current state with the state further back in time.^38^ To address this issue, LSTM networks harness a cell state **C**_*t*_ to store the long-term information, as shown in Figure 1b. As with RNNs, the information flows through time in LSTM networks, but both short-term and long-term memory are carefully carried via hidden state and cell state, respectively. Several variants of the LSTM unit have been developed^39,40^ since the LSTM unit was first proposed.^41^ The *standard* LSTM^39^ is used in the current study as it has been proved to outperform other variants^42^ and has been used extensively over a broad range of applications.^43,44^

The core advantage of LSTM is its ability to control information deletion from or addition to the cell state. As depicted in Figure 2a, the cell state runs through the LSTM unit at the top of the diagram with some minor interactions, indicating that the information stored in the cell state could flow along with slight or no changes. These interactions are carefully regulated by different gates.^41^ The role of the first one, the *Forget Gate*, is to decide how much information from **C**_*t*–1_ should be discarded. This decision is made by a Sigmoidal activation function σ(*x*) = 1/(1 + *e*^–*x*^) looking at the previous hidden state, **h**_*t*–1_, and the current input, **x**_*t*_
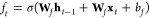
1

The subsequent *Input Gate* determines the new information to be memorized in **C**_*t*_
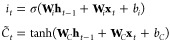
2

Herein, the candidate cell state, , is introduced to describe the current input. Later, the cell state is updated by
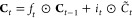
3where ⊙ denotes the Hadamard product, i.e., element-wise multiplication [*A* ⊙ *B* gives a matrix of the same dimension with *A* and *B*, where elements are given by (*A* ⊙ *B*)_*ij*_ = (*A*)_*ij*_(*B*)_*ij*_] of vectors and matrices. Lastly, the *output gate* is applied to settle the final output of the LSTM cell
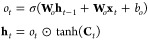
4which contains the **h**_*t*_, a cache of the recent information from **h**_*t*–1_, and long-period aspects from **C**_*t*_. In the equations above, **W** and *b* are trainable parameters of the LSTM unit, representing the weight vectors and the offset term for different gates, respectively. These equations are computed for each time-step and hence with a subscript *t* denoting the time-step. As described above, LSTM is capable of learning aspects in long-time sequences and thus has been used in modeling dynamical systems.^20−22^ A detailed architecture of the model employed in the current study will be presented in the following section.

More recently, Cho et al. proposed a new type of gated RNN, GRU, reducing the number of manipulating gates to two, which is shown to perform comparable to the LSTM. As shown in Figure 2b, the two gates are called a *Reset Gate*, *r*_*t*_ and an *Update Gate*, *z*_*t*_, which could be written in the form similar to those in the LSTM unit
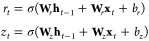
5

In addition, GRU RNN has only one hidden state as the traditional RNN presented as
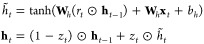
6

With these equations, GRU saves the one gate and one internal memory state and thus the associated parameters to be computed. As a result, its training time is expected to be shorter than that for LSTM. Herein, we compared the performance of both architectures briefly, in addition to the computation time and memory occupation during the training, and the corresponding results are deferred to the section Results and Discussion.

## Methodology

The computational framework presented in this work adheres to a three-stage workflow consisting of (1) data acquisition, (2) data reconditioning, and (3) model training, deployment and explainability, as illustrated in Figure 3 and detailed throughout this section. At its core, we constructed a multivariate RNN capable of carrying out multistep predictions of the temporal evolution of key multidimensional dispersion performance metrics, while remaining agnostic to the specifics around the mixing process itself. It is worth noting that each mixing device considered herein (i.e., static and stirred mixers) was trained separately. This separation stemmed from a disparity in the time-series dimensions between mixing systems (stirred vessel cases have three times as many time-steps in comparison), which poses a challenge for the model to handle, as we will discuss further in this section.

### Data Acquisition

#### Problem Statement: CFD Simulations of Mixing Systems

This study utilizes high-fidelity CFD data of two widely employed mixing systems in the industry: stirred tanks and static mixers handling two-phase L–L flows. Each mixer operates in a completely different flow regime, with the former handling transitional/turbulent flows (Re = ρND_*r*_^2^/μ ≈ [9000, 18,000]) and the latter operating under laminar conditions (Re = ρ*U*_*r*_*D*_*r*_/μ = 1.63). For brevity, specifics on the problem formulation of each system will not be described herein, but readers are encouraged to refer to previous publications detailing the geometrical and operational specifications, fluid properties, numerical considerations (e.g., grid refinement), and validation.^28−31^ The extracted data sets comprise multidimensional time-series data encompassing three key metrics integral to the dispersion performance: interfacial area growth (IA), drop count (ND), and DSD, calculated as the approximate volume of cells resolving a fully detached structure or “drop”. The choice of these parameters capitalizes on the explicit and robust nature of the interface-tracking scheme [level-contour reconstruction method (LCRM)] embedded in the CFD code used, which furnishes a more accurate and well-resolved representation of the intricate interfacial dynamics compared to other traditional schemes (e.g., level-set methods).^45^

A comprehensive set of 43 simulations was considered: 14 cases involved stirred vessels, exploring various rotational speeds (*N*_rot_) and surfactant profiles, while the remaining 29 cases focused on static mixers, investigating different inlet configurations, geometry arrangements, and types of surfactants. While the former 14 cases are divided between clean (varying *N*_rot_) and surfactant-laden systems, the latter 29 overlap the inlet setup and geometry arrangement with clean and contaminated scenarios. The specific parameters, combinations, and value ranges explored for each set of cases are detailed in Table 1. The split between training, validation, and test cases for static and stirred mixers, respectively, was set as (16, 5, 8) and (9, 2, 3), achieving a rough 75–25% split between training and testing sets. The case selection for each set was done manually to guarantee a sufficiently diverse split to capture all features considered (e.g., surfactant activity, operational conditions, etc.). For instance, all three data sets in the static mixer scenario contain cases with either surfactant activity, varying inlet morphology, or different mixer arrangement (as seen in the legends for Figures 9–11). Furthermore, validation and test cases attempt to cover a broad spectrum of features by including both high and low-solubility surfactants, as well as clean cases with standard and alternative mixer arrangements.

**Table 1:** Simulation Cases Considered in This Study, Categorized into Three Broad Groups According to the Focus of Each Study: Varying Surfactant Properties, Modifications in the Operating Conditions, and Different Mixing Element Arrangements, Specific to Static Mixers

mixer/case	stirred mixer	# cases	static mixer	# cases
surfactant-laden			**3-drop inlet**	
	Bi = [0.001,1]	5	Bi = [0.01,1]	3
	β = [0.5,0.9]	3	β = [0.3,0.9]	3
			Da = [0.01,1]	3
operational configuration	**rotational speed (Cl)**		**clean premix (pm)**	
	*N*_rot_ (Hz) = [5,10]	6	inlet = [coarse, fine, 3-drop]	3
			**surfactant-laden (coarse)**	
			Bi_pm_ = [0.01,0.1]	2
			β_pm_ = [0.6,0.9]	2
			Da_pm_ = [0.1]	1
geometrical arrangement	N/A	N/A	**clean**	
			coarse pm = [Alt1, Alt2, Alt3]	3
			fine pm = [Alt4]	1
			**surfactant-laden**	
			3-drop inlet (Alt 4)	
			β_alt4_ = [0.3,0.9]	3
			Bi_alt4_ = [0.01,1]	3
			premixed inlet	
			coarse_β=0.9_ = [Alt1, Alt4]	2

**Figure 4:**
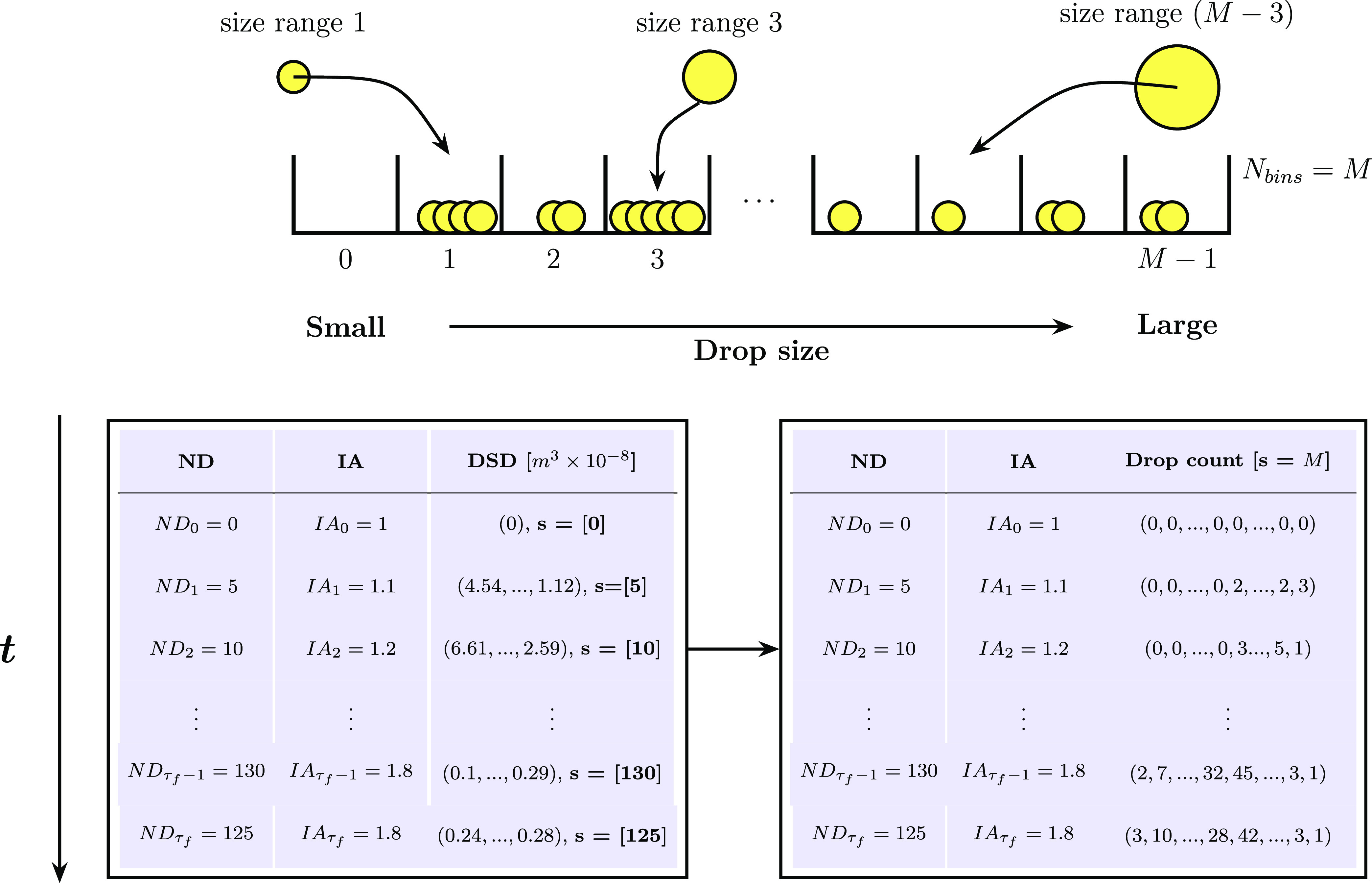
Schematic detailing DSD binning transformation. Top diagram showcases the drop size sorting and counting process in each size range, while the bottom tables show the transition from variable-sized volume lists (*s* = ND_*t*_) to discrete drop counts in a fixed number of bins (*s* = *M*).

**Figure 5:**
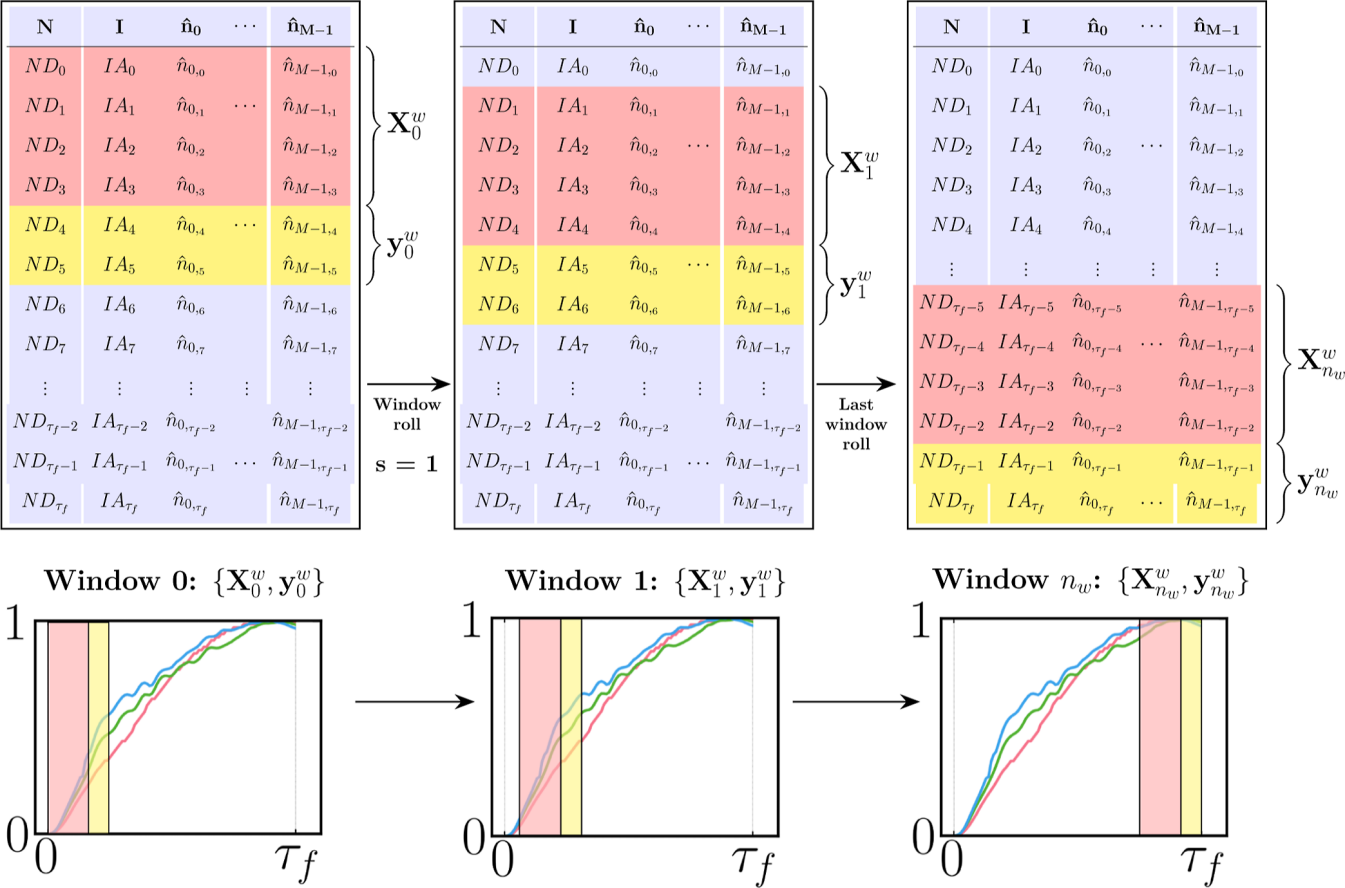
Schematic representation of the time-domain S2S windowing procedure implemented in this study. The top part provides a tabular representation of the windowed data sets, while bottom figures illustrate the rolling window procedure on top of a generic feature. Here, the window size is set to *s*_w_ = 6, where the number of input and target steps is *n*_*i*_ = 4 and *n*_*k*_ = 2, respectively. The window is shown to be rolled with a stride of *s* = 1.

**Figure 6:**
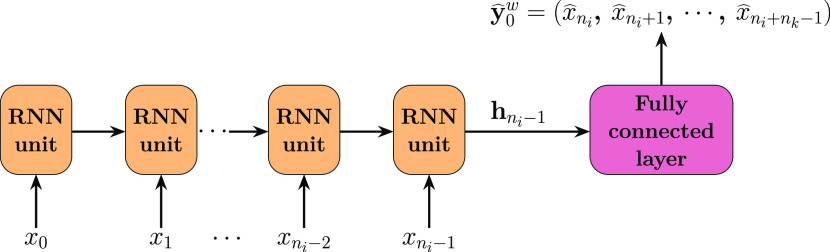
Diagram of an RNN-FC neural network architecture, where each unit can be either an LSTM or GRU cell.

**Figure 7:**
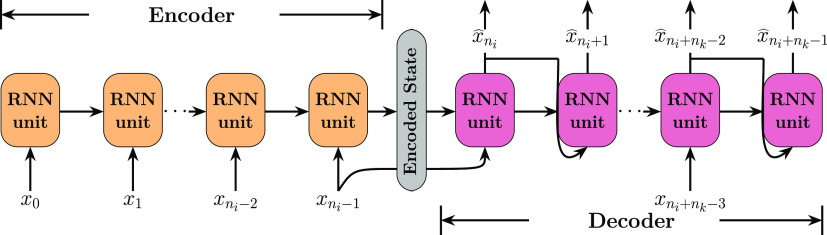
Diagram of an RNN encoder–decoder (ED) architecture, implementing mixed teacher forcing training. As for Figure 6, the RNN units can either be LSTM or GRU cells.

**Figure 8:**
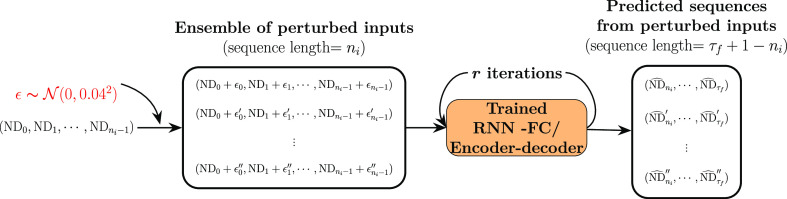
Diagram showcasing the procedure of ensemble-based uncertainty quantification. Take ND as an example, perturbations are introduced to the input sequence by adding noises, ε, drawn from a Gaussian distribution, , at each time-step, giving rise to the ensemble of perturbed inputs. All of the ensemble members are fed into the trained model, which progressively produces the ensemble of predicted sequences (with a sequence length of τ_*f*_ + 1 – *n*_*i*_). *r* is the number of iterations determined by the target sequence length, *r* = (τ – *n*_*i*_)/*n*_*k*_.

**Figure 9:**
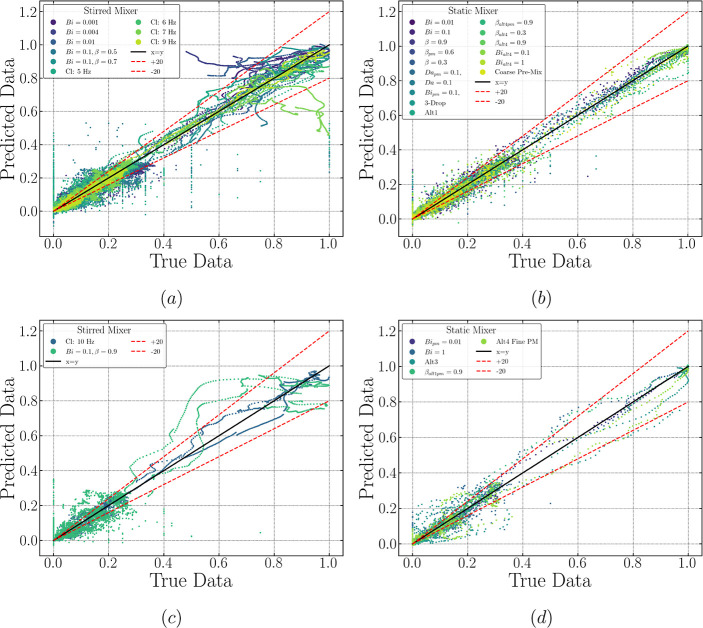
LSTM-FC predicted vs true data error dispersion plots for all 12 features considered in this study. A ±20% deviation area is included. Subfigures to the left (a,c) showcase training and validation data for stirred mixers, while those to the right (b,d) illustrate training and validation data for static mixers, respectively.

**Figure 10:**
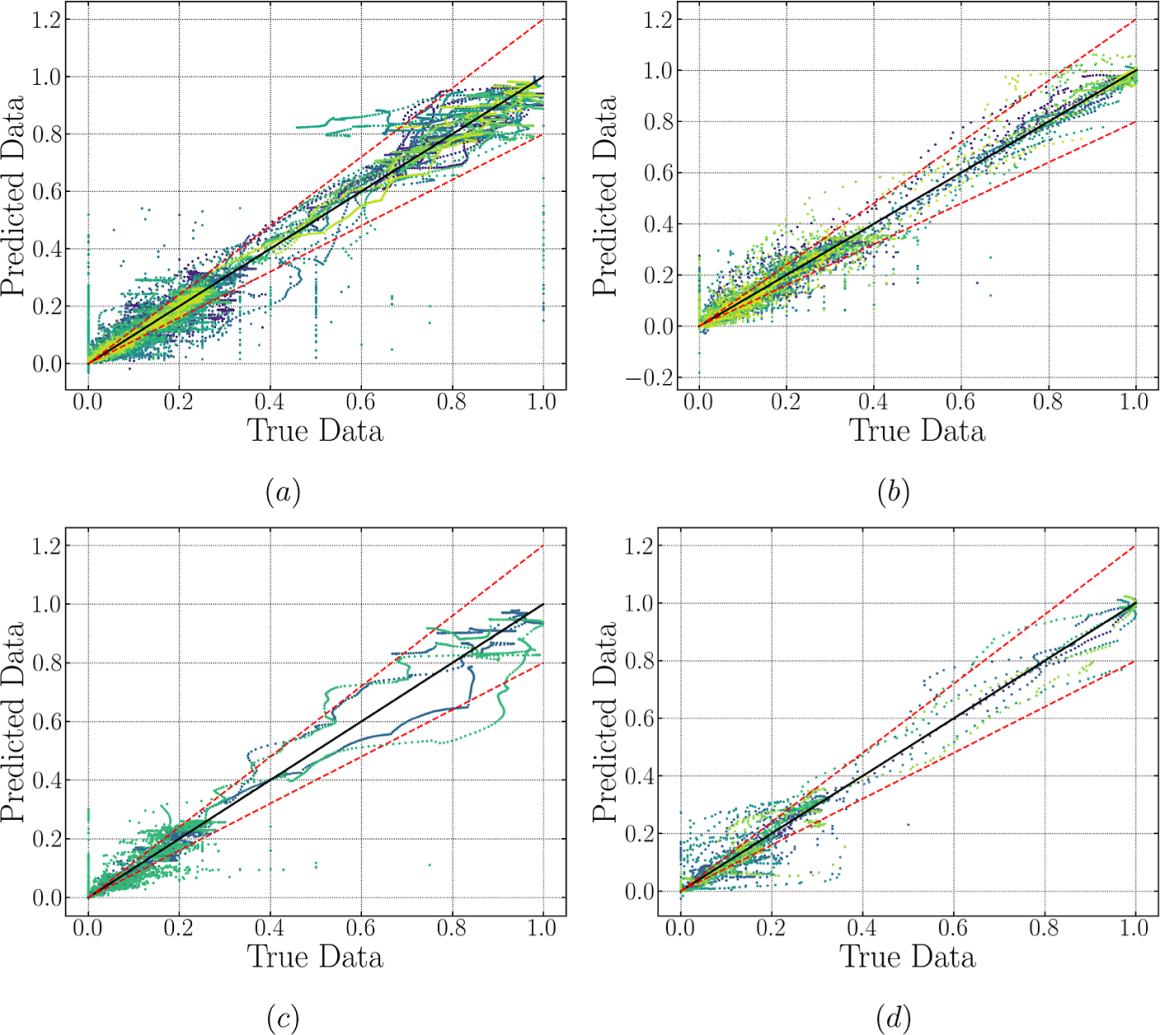
LSTM-ED predicted vs true data error dispersion plots for all 12 features considered in this study. A ±20% deviation area is included. Subfigures to the left (a,c) showcase training and validation data for stirred mixers, while those to the right (b,d) illustrate training and validation data for static mixers, respectively. Plot legends are shared with Figure 9, and thus not included here to avoid redundancy.

**Figure 11:**
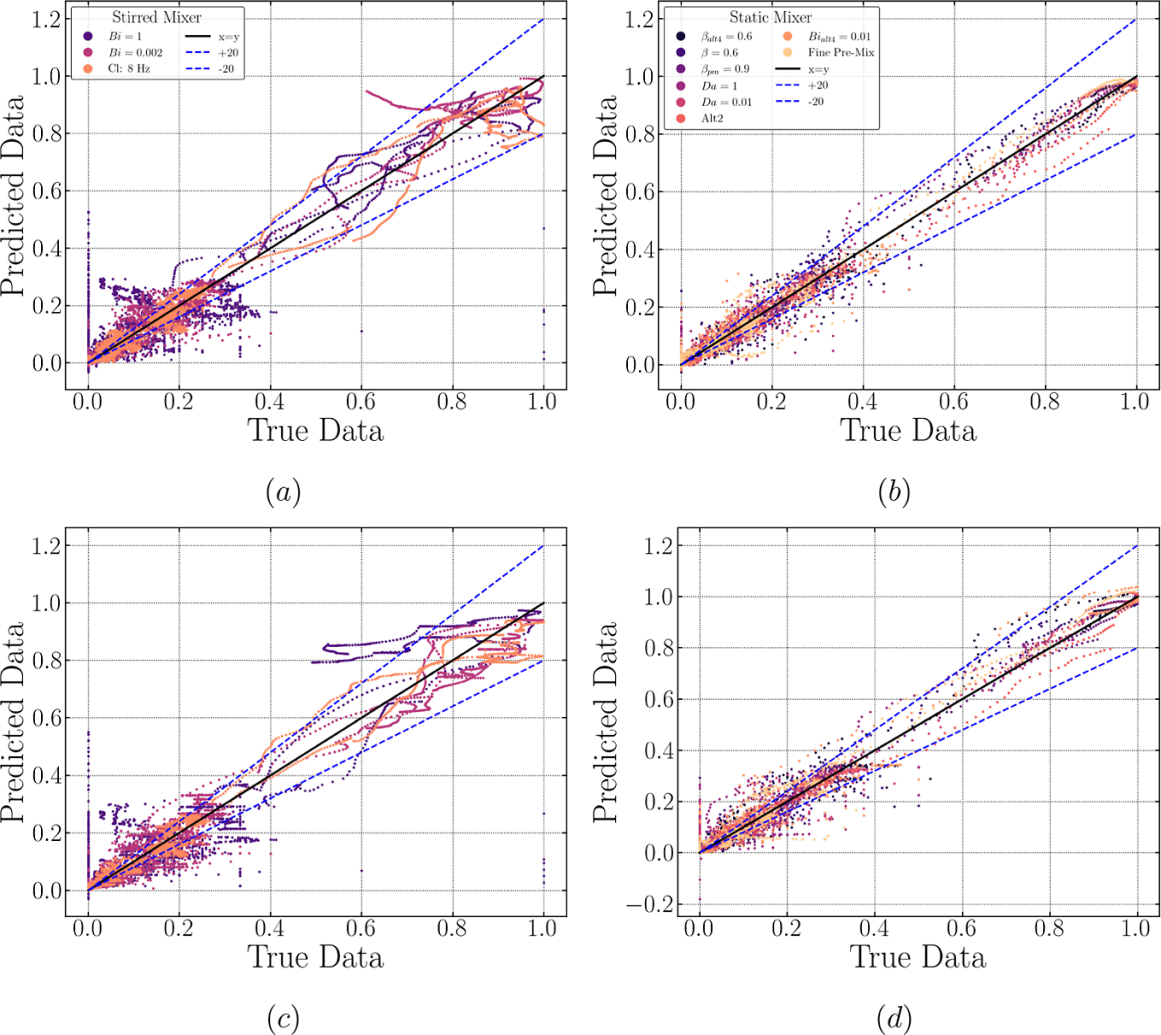
Predicted vs true data error dispersion plots for testing data sets, with a ±20% deviation region included. Subfigures (a,c) showcase rollout prediction data dispersion for the stirred mixer via FC and ED architecture, while subfigures (b,d) illustrate rollout prediction data for the static mixer via FC and ED, respectively.

Simulations were carried out using in-house code BLUE,^32,45,46^ which considers a three-dimensional single-field formulation of the Navier–Stokes equations in a Cartesian domain
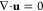
7
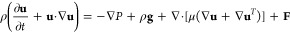
8where *t*, *P*, **u**, **g**, and **F** denote time, pressure, velocity, gravity, and a local surface force. For clean systems, this term follows a hybrid formulation of the form
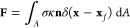
9where σ is a constant interfacial tension coefficient, κ is the interface curvature, and the 3D Dirac delta function δ(**x** – **x**_*f*_) is set to 0 everywhere and unity at the interface, which is located at **x** = **x**_*f*_.^31^ A numerical Heaviside function, , is used to define density and viscosity fields throughout the domain, which are respectively given by
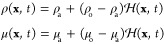
10

This Heaviside function is defined to have a value of 1 for the oil phase (subscript o) and 0 for the aqueous phase (subscript a) and is generated through a vector distance function from the interface φ(**x**) and solved numerically with a smooth 3–4 grid cells transition.^30,46^ Additional terms are added to the formulation in eq 8 when dealing with stirred tanks. First, a Smagorinsky–Lilly LES turbulence model is implemented, adding the filter  to the dissipation term, and second, a direct forcing method is added with the inclusion of a fluid–solid interaction force, **F**_fsi_.^28^

In the presence of surfactants, the force **F** is decomposed into its normal (σκ**n**) and tangential components (∇_s_σ), as shown in eq 11
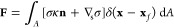
11where κ denotes the interface curvature, ∇_s_ stands for the surface gradient operator, and **n** is the normal unit vector pointing away from the interface. In surfactant-laden cases, σ is no longer constant but is modeled through a Langmuir EoS of the form , where Γ refers to the surfactant surface concentration. The chemical nature of the surfactant is parametrized by the following dimensionless numbers
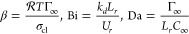
12where β, Bi, and Da stand for the elasticity, Biot, and Damkohler numbers, characterizing surfactant “strength” (interfacial tension sensitivity on concentration), desorptive capability vs convective surface transport, and adsorption depth into the bulk, respectively.^31^ These parameters are used to label the surfactant-laden cases employed herein and broadly describe the nature of each surfactant modeled (e.g., highly/weakly adsorptive/desorptive, etc.). Further details into the equations governing surfactant transport and the specifics behind the interface-tracking algorithm (i.e., LCRM) are found in previous publications.^29,31,32,45^

#### Data Preprocessing: Scaling, Smoothing, and DSD Binning

The initial set of features considered in this study consists of two scalar quantities, namely, IA and ND, and a variable-sized list of scalars (i.e., drop volumes) representing the DSD. To maintain dimensional consistency in the former two features across all cases sharing the same mixing system, a postsequence truncation is implemented up to a final time-step τ_*f*_ (see table illustrations in Figure 4), which corresponds to the length of the shortest sequence within a given mixer’s pool of cases. However, the length of the DSD feature remains dependent on the number of drops at each time-step *t* (ND_*t*_), which is inherently linked to the governing physics of each case. This inconsistency effectively renders a three-feature input sequence with a case-specific, uneven feature size of (1, 1, ND_*t*_) per time-step *t*.

Handling input sequences with varying feature lengths represents a challenge for the network architectures implemented here, as RNNs and in particular standard LSTM networks are designed to have a fixed topology a priori (i.e., invariant parameter size),^47^ thus requiring to be fed with fixed-sized input sequences of equal feature lengths.^48,49^ This fixed-size requirement aims to prevent potential issues such as information loss and limitations on the model’s capability to learn meaningful long-term dependencies. Previous works have implemented techniques to circumvent this flaw, such as padding, truncation, or “attention” mechanisms. The former two are common approaches implemented in text recognition^50^ and image processing,^49^ where the length of the longest (padding) or shortest (truncation) sequence is set as the standard, and each sequence is either filled with zeros or has data removed accordingly.^49^ Despite the benefits of longer padded sequences (Khotijah et al. demonstrated consistently higher model accuracy when handling longer sequences) or the easiness of dealing with truncating data sets, other studies have suggested alternatives (e.g., nearest neighbor interpolation) arguing that padding can be computationally demanding and naive truncation methods can lead to critical information loss.^51^ More sophisticated methods such as *attention layers*(52) have shown remarkable potential in adequately filtering relevant input subsets. Still, they have been seen to fail when handling long-time-step predictions.^53^

Based on the above, we explored an application-specific approach to address the fluctuating DSD feature size without compromising the integrity of the data sets or substantially increasing the model’s complexity and resource requirements. Our proposed method, depicted in Figure 4, consists of transforming drop volume data into discrete drop counts for different size ranges. These counts can be then partitioned into a fixed number of *M* bins ([*B*_0_, *B*_*M*–1_]), serving as individual features which monitor the number of drops entering or exiting a given size range over time. These features are subsequently scaled between 0 and 1 through a normalized probability density estimation procedure, using the count of each bin as a density estimate as showcased in eqs 13 and 14 for a bin *B*_*l*_ ∈ [*B*_0_, *B*_*M*–1_] at a time *t*
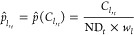
13
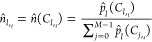
14where  and  denote the probability and normalized density estimators, respectively, for a drop count value  corresponding to *B*_*l*_ at time *t*; ND_*t*_ stands for total drop count at time *t*, and *w*_*l*_ denotes the width of *B*_*l*_. Our proposed approach introduces *M* additional features to the RNN, thus increasing the computational resources needed for training. However, the resolution of the DSD (*M*) can be adjusted depending on the system studied, thus acting as a refinement parameter that balances accuracy and computational cost. In this work, *M* was initially set to 20 and 12 for the stirred and static mixing cases, respectively, aiming to provide sufficient resolution for the DSD data based on statistical analyses conducted in previous publications.^29,31^ To eliminate outliers and uninteresting features (i.e., bins that mostly remain as 0 throughout the time domain), *M* was ultimately cut to 10 bins for both mixers (*B*_0_ – *B*_9_), dropping the boundary bins at each end of the size range proportionally, yielding 12 features overall for the RNN to handle.

**Figure 12:**
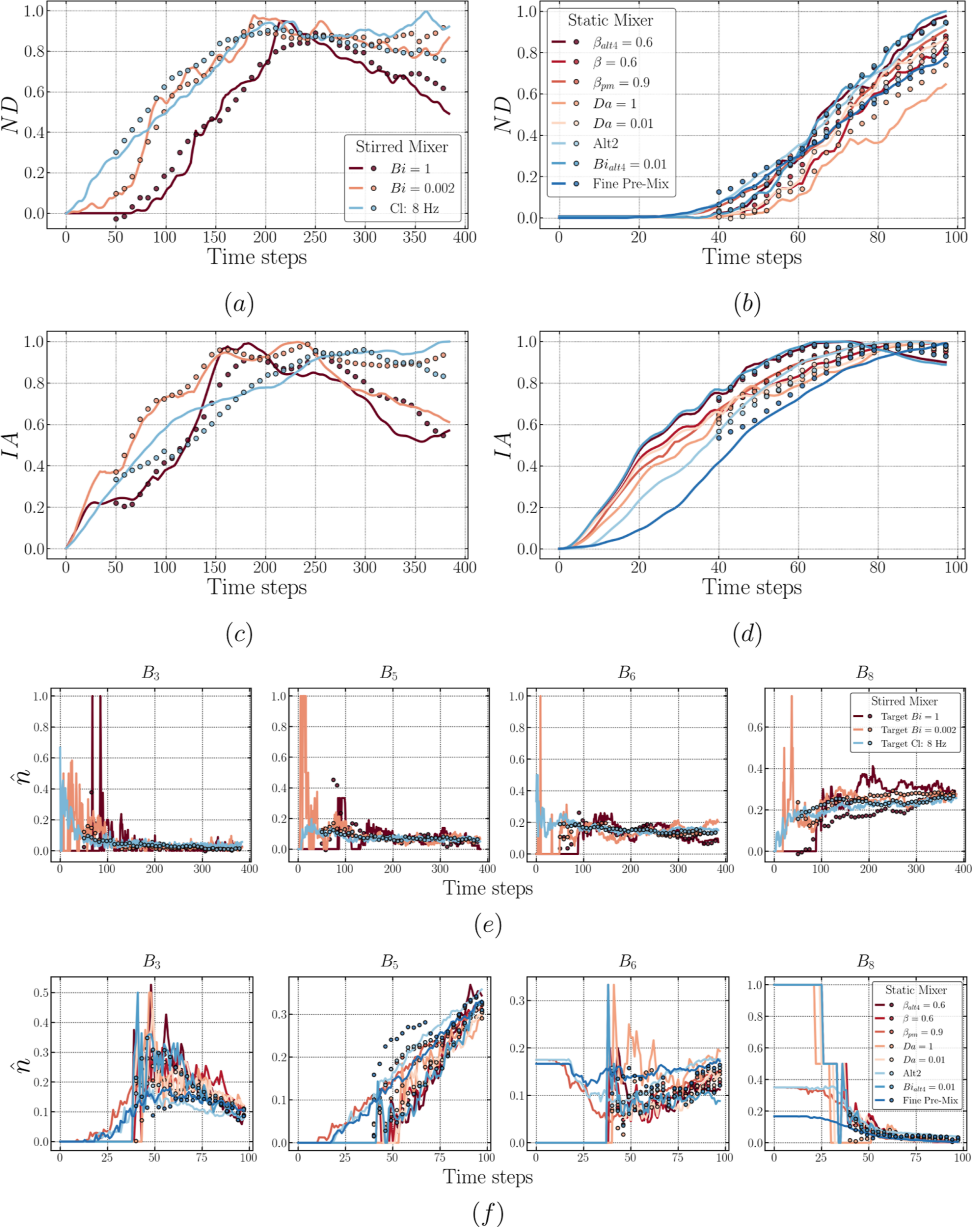
Plots comparing the model target sequences (lines) and predicted sequences via the rollout procedure (dots) of LSTM-FC for both mixers [left plots (a,c) correspond to the stirred mixer, (b,d) correspond to the static mixer, with features ND and IA in the top and bottom, respectively]. The results of predicted drop size distribution are exemplified using *B*_3_, *B*_5_, *B*_6_, and *B*_8_ for both mixers (e,f).

**Figure 13:**
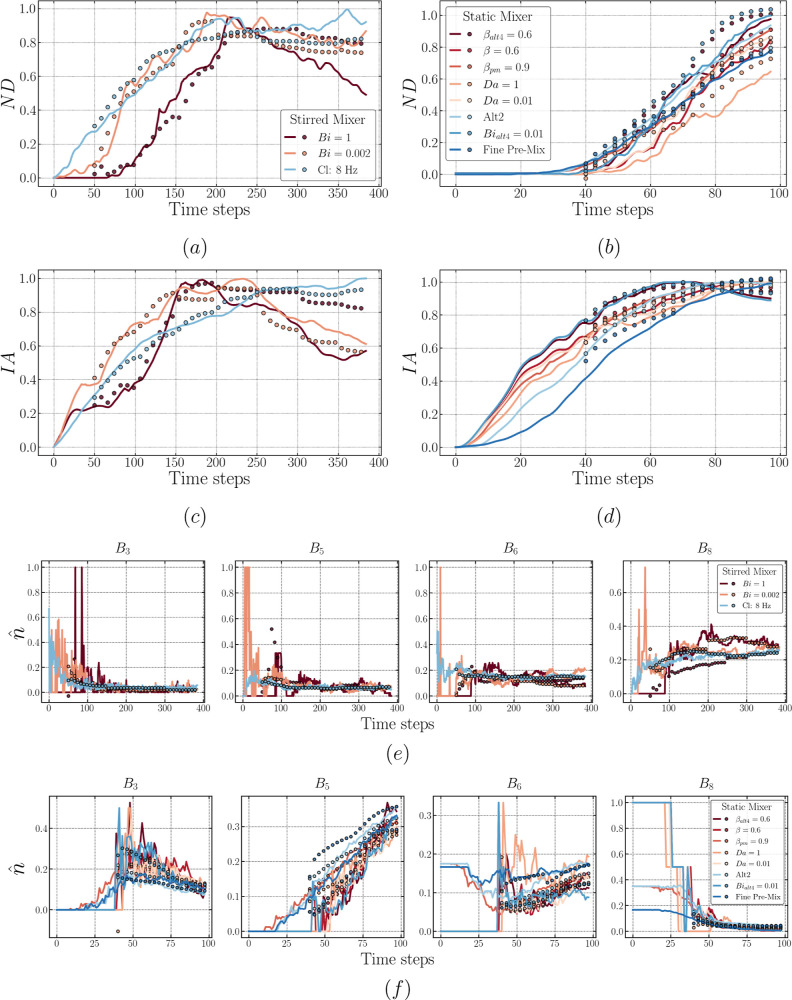
Plots comparing the model target sequences (lines) and predicted sequences via the rollout procedure (dots) of LSTM-ED for both mixers [left plots (a,c) correspond to the stirred mixer, (b,d) correspond to the static mixer, with features ND and IA in the top and bottom, respectively]. The results of predicted drop size distribution are exemplified using *B*_3_, *B*_5_, *B*_6_, and *B*_8_ for both mixers (e,f).

Finally, the ND and IA features were scaled and smoothed individually to mitigate potential biases arising from their substantially different scales [∼*O*(10^2^) vs ∼*O*(1)] and the sharply fluctuating trend observed for the ND data. The scaling procedure was applied to each individual feature on a case by case basis, through a linear sklearn MinMaxScaler method, restricted to a range of [0, 1]. Subsequently, three smoothing techniques were tested, namely, moving average, Savitzky–Golay filter, and Locally Weighted Scatterplot Smoothing, or “Lowess”. Following an initial visual examination, the Lowess method, with a fraction of δ = 0.06, and the Savitzky–Golay filter, with a window size of 5 and a third-order poly fit, yielded the best behavior for the stirred and static mixer data sets, respectively. This selection was based on the filter’s ability to minimize noise in the ND feature while preserving other essential ND and IA characteristics (e.g., inflection points). The former method conducts a weighted linear regression at each point using a cubic weight function *W*(*x*) = (1 – |*x*|)^3^, based on the nearest *N* × δ data points. Meanwhile, the latter performs a *k*-order polynomial fitting within a specified window length based on the least-squares principle.^54^ The normalized density estimations from the binned DSD data were not subjected to smoothing due to their extremely noisy behavior (see Figures 12 and 13e,f). The methods probed herein proved insufficient when attempting to retain the most relevant temporal trends, particularly for the boundary bins (i.e., largest or smallest sizes).

### Data Reconditioning: Augmentation through Windowing

Data augmentation is the process of generating synthetic data that incorporates the existing knowledge about invariant properties of the original data against specific transformations. This procedure lowers the risk of model overfitting and enhances its accuracy and generalization capabilities by providing a diverse yet realistic data set.^55^ Data augmentation has been actively probed for classification models in the field of computer vision (e.g., flipping, rotating, scaling images, and adding Gaussian noise/distortion), but has been less explored in time-series scenarios given their vulnerability to transformation procedures.^55,56^ Methods used for image data augmentation are not well generalized with time-series data, since some of their intrinsic properties (i.e., temporal dependency) are not fully leveraged by such methods and there is no assurance that the meaning of the raw data will remain unchanged.^56,57^

While it is possible to perform a frequency domain transformation to the time-series data to facilitate the application of some of these methods, additional complications emerge when dealing with complex or inherently intertwined multivariate time series data,^57^ as is the case for the features studied herein. In such scenarios, it is advised to develop a tailored augmentation methodology that conserves the original data semantics.^56^ Considering the above, and given the limited number of simulation runs available for training, we devised a basic time domain sequence-to-sequence (S2S) rolling window augmentation technique, which aims to expand the training data available while preserving their original semantics. Let us first examine the characteristics of the data and the training procedure without augmentation.

Let **X** be the original time-series array of a case under consideration with dimensions *D* = (τ_*f*_ + 1, *M* + 2), where τ_*f*_ + 1 and *M* + 2 correspond to the total number of time-steps and features, respectively. A representation of the time-series array is shown in eq 15
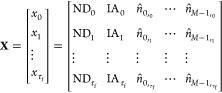
15where . The RNN training requires the original time series, **X**, to be divided into input (**x**) and target (**y**) arrays, the latter acting as the ground truth for the model to evaluate its predictions. Accordingly, we define *n*_*i*_ as the number of input steps aimed to be fed into the RNN and *n*_*k*_ as the number of target steps to predict into the future. Assuming τ_*f*_ = *n*_*i*_ + *n*_*k*_ – 1, we can then define subsets , denoting the input and target sequences from the original time-series, with dimensions *D*_**x**_ = (*n*_*i*_, *M* + 2) and *D*_**y**_ = (*n*_*k*_, *M* + 2), respectively, as given by eq 16.
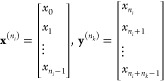
16

This method would render an insufficient 10 or fewer training data sets for either mixing system (1 set per case). Therefore, the case-wise windowing approach implemented here seeks to augment the number of input-target sequence pairs  from a single data set, as explained below and shown schematically in Figure 5:

1. **Window creation:** The aim is to split the original data set, **X**, into a set of *n*_w_ + 1 input and target sequences, denoted as , with arbitrarily reduced dimensions *D*_**X**^w^_ and *D*_**y**^w^_, following the same definitions introduced earlier for *n*_*i*_ and *n*_*k*_, but now considering *n*_*i*_ + *n*_*k*_ ≪ τ_*f*_. The input/target pair is referred to as “window”, and its size along the temporal axis is defined as *s*_w_ = *n*_*i*_ + *n*_*k*_. The procedure starts by building window 0 from *t*_0_ to , as seen in Figure 5. This can be expressed as **X**_0_^w^ = **X**[0: *n*_*i*_ – 1,:], **y**_0_^w^ = **X**[*n*_*i*_: *s*_w_ – 1,:].
2. **Window rolling:** Window 0 is then rolled forward to generate window 1: X_1_^w^ = **X**[1: *n*_*i*_,:], **y**_1_^w^ = **X**[*n*_*i*_ + 1: *s*_w_,:], where the rolling obeys a user-defined stride *s*, which was set to *s* = 1 in this work (see Figure 5). In general, the window for the next iteration *j* + 1 with *s* = 1 is **X**_*j*+1_^w^ = **X**[*j* + 1: *j* + *n*_*i*_,:], **y**_*j*+1_^w^ = **X**[*j* + *n*_*i*_ + 1: *j* + *s*_w_,:].
3. **Data stacking:** The rolling procedure in step 2 is repeated *n*_w_ times, where *n*_w_ is determined by the expression *n*_w_ = τ_*f*_ + 1 – *s*_w_, taking *s* = 1. After each roll, the generated input/target sequences {**X**_*j*_^w^, **y**_*j*_^w^} in each window are stacked separately into new tensors **W**_**X**_, **W**_**y**_, respectively, as shown in eq 17:

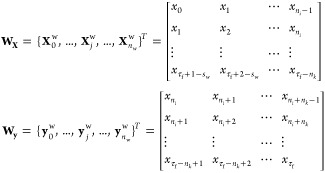
17where , , and . The augmentation procedure described above does not incur data leakage problems given the specific data loading and training procedure implemented in this study, as we will discuss in the upcoming subsection.

In this work, *n*_*i*_ and *n*_*k*_ constitute user-defined parameters in the RNN architecture. These parameters serve a dual function: instructing the network on the number of input and output steps it will handle and constrain the number of windows created. On the other hand, τ_*f*_ is an intrinsic characteristic of the original time-series data set. It is worth mentioning that *n*_*i*_, *n*_*k*_, and thus *n*_w_ may affect the outcome of the RNN training and rollout procedure, as they determine how many times the model iterates over its output, and consequently how much error propagation the results are exposed to (as we will discuss further on). However, this aspect of the model was not treated as a tunable hyperparameter as it would entail reprocessing the entire raw data set for every model training variation tested. Consequently, this would impose a substantial increase in computational expenditure. The values for (*n*_*i*_, *n*_*k*_) were set to (40, 30) and (50, 50) for the static and stirred mixer, respectively. This yields *n*_w_ = (29, 286) windows per case considering each mixer’s respective τ_*f*_ [refer to section Prediction via Sequence Generation (Rollout)]. Consequently, for the static and stirred mixer respectively, 464 and 2574 windows were allocated for the training set, whereas 145 and 572 windows were generated for the validation set.

### Model Training, Deployment, and Explainability

#### RNN Model Architectures and Training Procedure

As mentioned at the onset of this section, we aim to construct a multivariate RNN network to perform multistep time-series predictions. The framework implemented herein was built using Python package PyTorch, since its framework inherently supports multistep input/target sequences with a constant number of features. The first architecture tested in this study is a simple network composed of a single RNN layer (with either LSTM or GRU units) and one fully connected layer (RNN-FC), where the latter reads and reshapes the hidden state, **h**_*t*_, from the former to yield a predicted sequence. As seen in Figure 6, the RNN layer processes a complete input sequence of *n*_*i*_ elements sequentially. Each element, , contains all *M* + 2 features per time-step, as previously introduced. Subsequently, the hidden state from the last time-step, , is fed into the FC layer to generate the predicted output sequence. This output sequence comprises *n*_*k*_ elements, each holding the same dimensions as those assigned to the elements within the input sequence. Connecting with the prior discussion, Figure 6 showcases the RNN-FC network handling Window 0, where it reads the first element **W**_**X**_^0^ = {**X**_0_^w^} as an input sequence and predicts , corresponding to the target sequence pair **W**_**y**_^0^ = {**y**_0_^w^}.

Despite the simplicity of the previous neural network, more specialized architectures have been specifically designed to tackle multistep forecasting. A common example is the RNN encoder–decoder architecture (RNN-ED) as coined by Cho et al., also commonly referred to as a multistep RNN, which comprises two RNN sublayers. The input layer, referred to as the *Encoder*, derives a compressed representation of the input sequence, referred to as the encoded state. The second sublayer, referred to as the *Decoder*, interprets the encoded representation and exploits it to generate the predicted target sequence. Unlike the previous model, predictions in this approach are computed sequentially from the hidden states of the RNN units themselves, instead of coming from a single reshaping layer at the end of the network. As depicted in Figure 7, the last element of the input sequence, , and the encoded state of the last RNN unit in the encoder layer (corresponding to its hidden and cell state, , , respectively) are sent into the decoder layer, which is the one responsible for generating a time-step prediction per RNN unit.

A noteworthy advantage of this more intricate architecture over the RNN-FC is its flexibility when it comes to training methodologies. First, the predicted output of each RNN unit in the decoder layer can be recursively fed back into the following unit, until an output of the desired length is generated; this process is generally referred to as *recursive* prediction. Alternatively, and similar to the encoder layer, true/target data can be exclusively fed into the decoder units to make computations, which is known as prediction via *teacher forcing*. Finally, both predicted outputs and true data can be alternately fed throughout the decoder layer; in such a scenario, the model is said to be generated via *mixed teacher forcing*. For both *teacher forcing* and *mixed* methods, a *teacher forcing ratio* parameter or *t.f.* ratio can be defined. Its role is closely related to the batch-wise nature of the training methodology implemented in this study, as we will explain in further detail up next. The *t*.*f*. ratio in *mixed* predictions determines the balance between using true data and predicted outputs in the decoder layer of the model per batch. On the other hand, the *t*.*f*. ratio for *teacher forcing* predictions dictates whether batches are exclusively fed with ground truth or predicted outputs. Therefore, in the *mixed* method, batches receive a combination of ground truth and predicted outputs, while in the *teacher forcing* approach, batches are exclusively fed with either true or predicted data. Furthermore, a Boolean *dynamic teacher forcing* (*d.t.f.*) parameter can be introduced. When this parameter is set as “True”, the teacher forcing ratio is gently reduced at each epoch (i.e., one complete pass of the training data set during model training). In this way, the model is trained to learn patterns from the target data at the early times, but it gradually acquires knowledge from its output, relying more on it to generate future predictions.

In this study, a shuffled batch-wise training methodology was adopted for both RNN architectures. This procedure consists of dividing the windowed tensors, **W**_**X,y**_, into smaller subsets or “batches”, thereby limiting the number of samples shown to the network during training before each weight updates and thus enhancing computational efficiency. In this approach, a set of input/target sequence pairs or windows, packed together into a batch , are indexed and randomly shuffled before being fed into the network. Batching and randomizing sequences enhance the model’s ability to generalize effectively across different data sets, improve its capability to discern common patterns, and prevent it from learning biases associated with the order of the data. Furthermore, for this application, window shuffling effectively mitigates the risk of data leakage by preventing the model from inadvertently learning dependencies between consecutive windows. This guarantees that the model encounters and treats each window as a unique individual instance.

In addition, a custom loss function was introduced during the training procedure, consisting of a standard mean square error loss function with an added penalty term that adopts the expression , where *w*_p_ stands for a user-defined penalty weight, ReLU(*x*) denotes the rectified linear unit activation function, defined as max(0, *x*), and  denotes a predicted sequence. This penalty term is meant to avoid negative predictions, which, in the context of this work, would yield nonphysical results (i.e., negative drop count). This is done by isolating and averaging all initially estimated negative values by the network and then adding a fraction of this average as penalty. In this way, loss decreases when negative predictions are minimized. Furthermore, two regularization terms *L*_1_ and *L*_2_, also known as Lasso and Ridge regressions, were added to the loss function to help manage overfitting. *L*_1_ introduces a penalty term based on the absolute value of the coefficient magnitudes, expressed as *l*_1_*∑*|β|, while *L*_2_ adds a penalty based on the square magnitude of the coefficients, given by *l*_2_ ∑β^2^. Both penalty terms are regulated by coefficients *l*_1_, *l*_2_, respectively.

In the final stage of model training, we introduced two key strategies: an early stopping procedure and a “*ReduceLROnPlateau*” scheduler. The early stopping mechanism continuously monitors improvements in the validation loss score and saves the best-performing model before any degradation occurs, effectively preventing overfitting. The scheduler optimizes model convergence by dynamically reducing the learning rate once the model performance stagnates (i.e., validation loss stops improving). It is worth noting that the scheduler implemented here uses the well-known adaptive moment estimation (Adam) optimizer,^58^, which is also employed for the backpropagation process throughout training.

#### Hyperparameter Tuning

Before initiating the training procedure to estimate the network’s learnable parameters (i.e., *weights* and *biases*), we carried out a comprehensive parametric sweep to optimize the framework’s user-defined parameters, also known as “hyperparameters”. As highlighted earlier, the input and target sequence sizes (*n*_*i*_, *n*_*k*_) were not included in this tuning step due to computational constraints. The remaining hyperparameters tuned in this study and shared by both network architectures and cell types include the hidden size (hidden state dimension), learning rate (step size for adjusting model weights during training), and batch size (number of windows fed during training before weight update), as well as loss-related weight parameters such as the custom penalty weight *w*_p_ and the *l*_1_ and *l*_2_ coefficients controlling the corresponding regression penalty terms described prior. On top of the above, the RNN encoder–decoder model considers two additional hyperparameters related to the training method adopted in the decoder layer, as explained previously, namely, the “Prediction type” and the *t.f.* ratio. In order to reduce the sample space explored during the tuning process and prioritize other more influential hyperparameters, the *d.t.f.* feature was fixed as “True” and the *recursive* training methodology was not included for the ED architecture.

We performed 8 separate exhaustive searches for each mixer, network and unit type combination in search for each specific case optimal configuration, representing a total of 1296 and 1536 parameter combinations for the FC and ED architectures, respectively. These parametric explorations were carried out using AI scaler package Ray Tune. The hyperparameter sample space explored is detailed in Table 2, along with each network best-performing model configuration. The parameter candidate values were selected based on early sensitivity trials and their relevance in the context of this study. For instance, a finer batch size sample space was explored given the relatively modest data set available for training and validation, which causes noise effects introduced from varying batch sizes to be more impactful on the generalization performance of the model. This is reflected in the widely different sizes obtained for each scenario. Other settings yielded expected results, such as larger hidden sizes for the stirred mixer given its substantially larger data sets, or nearly constant penalty weights, *w*_p_, given the similar nature of all features and data sets. Similarly, the *L*_1_ and *L*_2_ penalty terms were essentially deemed unnecessary for most models, as overfitting has already been mitigated in various other ways, as previously described (e.g., batch randomization). Only some FC architectures included an *L*_2_ regularization penalty, encouraging the model to use smaller weights to reduce the impact of problematic individual features (e.g., DSD bins) on the output. On the other hand, the LSTM-ED architecture always preferred a *mixed* training method with small *t.f.* ratios, implying that the model mostly does not rely on the ground truth to carry out predictions throughout the Decoder layer, but still prefers to have access at the early stages to improve its performance, rather than running exclusively on its output from the onset. This is not the case anymore when dealing with GRU units for ED networks, which seem to rely more heavily on the ground truth given the preference for a *teacher forcing* approach with high *t*.*f*. ratios, implying that the model requires a higher ratio of batches to use ground truth exclusively. Future work is suggested to explore the inclusion of the *d.t.f.* feature in the hyperparameter tuning exercise, as it might show the true dependency of the model on the ground truth.

**Table 2:** Hyperparameter Search Space and Best-Performing Network Configuration for Each Corresponding Architecture, RNN Unit Type, and Mixing System Studied^a^

hyperparameters	value ranges	network architecture			
		FC (LSTM/GRU)		ED (LSTM/GRU)	
		stirred	static	stirred	static
hidden size	64, 128, 256	(256/64)	64	256	(64/128)
learning rate	0.002, 0.005, 0.01	0.005	(0.01/0.002)	(0.002/0.005)	(0.005/0.002)
batch size	(8–40, 4)^b^	(36/12)	36	(40/36)	(24/20)
penalty weight (*w*_p_)	0.01, 0.1, 1, 10	0.1	(0.1/0.01)	0.1	0.1
prediction type	*t*.*f*., mixed	N/A		mixed	(mixed/*t*.*f*.)
*t.f.* ratio	0.02, 0.1, 0.2, 0.4	N/A		(0.1/0.2)	(0.02/0.4)
*l*_1_ coefficient (lasso)	0, 1 × 10^–5^	0	0	0	0
*l*_2_ coefficient (ridge)	0, 1 × 10^–5^	(0/1 × 10^–5^)	1 × 10^–5^	0	0

a Values inside brackets correspond to the same network architecture implementing either LSTM and GRU cells, respectively. If only one value is registered, it means that both models reported the same hyperparameter after tuning.

b Batch size range lies between 8 and 40, with a step of 4, rendering 9 parameter values.

#### Prediction via Sequence Generation (Rollout)

As explained earlier with the introduction of the windowing process and the model architectures, the RNN network is designed to map an input sequence of length *n*_*i*_ to an output sequence of fixed length, *n*_*k*_, where *n*_*k*_ + *n*_*i*_ ≪ τ_*f*_. Recall that our objective is to predict the entire temporal evolution of the dispersion features up to the final time-step, τ_*f*_, where simulations are terminated. To achieve this, a rollout procedure is implemented, wherein the trained model is iteratively reused until the desired time-step is reached. During each iteration, the previous output sequence is fed back into the trained model to predict the next *n*_*k*_ sequence. The number of iterations or “rollouts” is therefore determined by the length of the output sequence, following the expression *r* = (τ_*f*_ – *n*_*i*_)/*n*_*k*_.

The selection of *n*_*i*_ and *n*_*k*_ is not a trivial task since these two values determine the size and number of data samples available for the model to be trained with (refer to section Data Reconditioning: Augmentation through Windowing), which naturally has a direct effect on model performance and uncertainty. Considering the differing lengths of the time-series data sets for each simulation case, acknowledging that they have been truncated for each mixing system (τ_*f*_ = 385 and τ_*f*_ = 98 for the stirred and static mixer, respectively), the values for *n*_*i*_ and *n*_*k*_ were fixed to (50, 50) and (40, 30) for the stirred and static mixer, respectively. These values were subjected to an early sensitivity test, but a full-scale tuning process would be required to discover the optimal configuration for each mixing case study. Accordingly, the number of rollouts, *r*, is computed as 7 and 2 for the stirred and static mixer, respectively.

#### Uncertainty Quantification from an Ensemble of Perturbed Inputs

After adequately training the model, the next logical step is to estimate its prediction uncertainty. Regarding this, many researchers have contributed to understanding and quantifying the uncertainty in a neural network’s prediction (see the comprehensive review by Gawlikowski et al.).^59^ Yet, an approach to uncertainty quantification applicable in our current work is required to cope with the fact that the trained model iterates over its output numerous times, as described above through the rollout procedure. In other words, we require a method that is capable of tracking the evolving model uncertainty and granting us access to the model performance through the propagation. To achieve this, an ensemble-based procedure, adapted from the ensemble forecasting technique that has been extensively used in numerical weather prediction,^60−62^ is proposed herein.

As showcased in Figure 8, we start by introducing perturbations parametrized by ε to the input sequence. This involves adding noise, drawn from a distribution, to the feature values at each time-step (Figure 8 takes ND as an example). Herein, a Gaussian distribution with mean μ = 0 and standard deviation (referred to as std. dev. henceforth) σ_s.d._ = 0.04 is used, namely , such that the feature values in the perturbed input sequence represent a probable uncertainty arising from observed feature values at early dispersion. For instance, for a given mixing system, the measured dispersed drop count at early times could be a few drops off if measurement errors are considered. It is worth noting here that the added noise could lead to negative feature values in the perturbed input sequence, which would be physically unrealistic; nonetheless, the purpose of their inclusion is to examine the capability of the trained model to handle any type of disturbance in the input data. In this way, an arbitrary perturbed input ensemble containing 200 members is generated, which subsequently enters the trained model to produce an ensemble of corresponding predicted sequences.

With the sequences mentioned above, we first compute the std. dev*.* across the 200 ensemble members at each time-step using the empirical std. dev. expression per sample
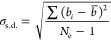
where *b*_*i*_, b̅, and *N*_e_ denote the *i*th member in the ensemble, the ensemble average, and the ensemble size (*N*_e_ = 200), respectively. Then, a prediction interval is calculated to give an overview of the range wherein the model prediction is likely to occur (with a 95% confidence) given the unperturbed input sequence, which could be written as
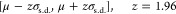


Lastly, we made use of the prediction interval to suggest a fair indicator of the model performance, namely, the absolute residual between the targeted perturbed evolution and its predicted counterpart from the trained model using the unperturbed input sequence. The corresponding results and relevant discussion are presented in the ensuing section.

## Results and Discussion

This section is subdivided as follows: first, a brief comparative evaluation of model performance is provided, examining accuracy and resource consumption metrics between both network architectures and for both mixing systems when utilizing either LSTM or GRU cells. From this, the top-performing RNN unit is chosen as the primary focus for the remainder of this section. Following this, we present a discussion on the chosen model’s generalization based on its performance on both training and validation data sets. Subsequently, we continue with an in-depth exploration of the chosen framework’s performance on the testing data sets. This involves a thorough analysis of the predicted temporal evolution for all features, namely, ND, IA, and DSD, across both mixing systems and network architectures. In particular, the DSD predictions are interpreted through selected bins, i.e., *B*_3_, *B*_5_, *B*_6_, and *B*_8_, for both mixers, containing the normalized density estimators, , introduced for the binning procedure in the section Methodology.

The drop size is recovered from the density estimators as a log-scale normalized drop volume, log_10_(*V*_d_/*V*_cap_), where *V*_d_ is the volume of the dispersed drop and *V*_cap_ denotes the volume of a spherical drop whose diameter corresponds to the capillary length scale, . Accordingly, the drop sizes covered in this work lie in a range of log_10_(*V*_d_/*V*_cap_) = [−4.5, 0.0] and [−7.25, −0.5] for the stirred and static mixer, respectively. Following the binning procedure described earlier, the actual volumes corresponding to each of the bins treated in this section are listed in Table 3. Lastly, the ensemble-based approach implemented to quantify the model’s uncertainty is showcased using features ND and IA from one testing case.

**Table 3:** Volumes of the Dispersed Entities Corresponding to the Size Ranges Presented in Figures 12 and 13

bin	stirred mixer		static mixer	
	λ_*c*_ = 0.0045[m]		λ_*c*_ = 0.0030[m]	
	bin edge	actual size	bin edge	actual size
	log_10_(*V*_d_/*V*_cap_)	[m^3^ × 10^–8^]	log_10_(*V*_d_/*V*_cap_)	[m^3^ × 10^–8^]
*B*_3_	[−3.50, −3.00]	[1.54 × 10^–3^, 4.86 × 10^–3^]	[−5.75, −5.00]	[2.54 × 10^–6^, 1.43 × 10^–5^]
*B*_5_	[−2.50, −2.00]	[0.02, 0.05]	[−4.25, −3.50]	[8.04 × 10^–5^, 4.52 × 10^–4^]
*B*_6_	[−2.00, –1.50]	[0.05, 0.15]	[−3.50, −2.75]	[4.52 × 10^–4^, 2.54 × 10^–3^]
*B*_8_	[−1.00, −0.50]	[0.49, 1.54]	[−2.00, −1.25]	[0.01, 0.08]

### Comparative RNN Unit Performance: LSTM vs GRU

As mentioned at the onset of this paper, two highly relevant and frequently employed RNN units (LSTM and GRU) were deployed to comparatively assess their performance and accuracy in an industrially relevant case study. The main difference between these two units is the absence of a cell state or long-term memory in the GRU structure, compared to LSTM. This is intended to diminish computational requirements (e.g., training time) without majorly compromising performance and the ability of the model to capture long-term dependencies.

Table 4 presents a comprehensive evaluation of all possible combinations between RNN architectures, units, and mixing systems. First, when looking at the overall RMSE, a slightly yet consistently lower set of values is obtained for LSTMs across both network architectures and mixing systems when compared to their GRU counterpart, indicating a generally better predictive capability. Similarly, for the overall *R*^2^ coefficient, the LSTM models always achieve higher values, albeit the difference is almost negligible in certain instances (e.g., <3% for static mixer cases). As expected, these observations suggest that LSTMs have a generally better read on the more intricate underlying patterns in the data and can capture data variability and long-term dependencies slightly more accurately than GRUs.

**Table 4:** Model Accuracy (for the Testing Set Only) and Computational Performance Metrics, Divided by Mixer Type and Sub-Divided by Network Architecture (FC/ED) and RNN Unit Type (LSTM/GRU)^a^

	stirred mixer				static mixer			
metrics	FC		ED		FC		ED	
	LSTM	GRU	LSTM	GRU	LSTM	GRU	LSTM	GRU
Accuracy								
RMSE (overall)	0.063	0.065	0.066	0.074	0.037	0.039	0.046	0.048
*R*^2^ (overall)	0.943	0.939	0.938	0.921	0.979	0.977	0.969	0.966
*R*^2^ (ND)	0.951	0.923	0.915	0.820	0.976	0.965	0.946	0.942
*R*^2^ (IA)	0.877	0.830	0.877	0.711	0.995	0.994	0.993	0.991
*R*^2^ (*B*_3_)	0.570	0.552	0.575	0.613	0.854	0.848	0.844	0.823
*R*^2^ (*B*_5_)	–0.051	–0.366	–0.042	0.591	0.931	0.915	0.896	0.848
*R*^2^ (*B*_6_)	0.600	0.567	0.416	0.579	0.531	0.573	0.665	–0.784
*R*^2^ (*B*_8_)	0.691	0.780	0.600	0.805	0.987	0.986	0.981	0.941
Performance								
tuning								
time [mins]	4915^+^	5726	7310	12,712*	614.900	595.482^+^	1366.947	1498.787*
peak memory [MB]	124.33	124.27	124.68	126.55	157.398	161.493	186.001	187.716
training								
time [mins]	59^+^	272	448	618*	7.234^+^	14.737	162.421*	117.984
peak memory [MB]	13.44	13.44	13.44	13.44	11.158	11.157	11.175	11.176

a Values marked with * and + correspond to the highest and lowest time taken for model tuning/training, respectively.

Similar conclusions can be drawn when looking at certain features individually, such as ND and IA, where the improvement attained when using LSTM instead of GRU units is significantly more noticeable, particularly for stirred mixers where a longer and more intricate time evolution unfolds for these features (e.g., 10–20% higher *R*^2^ coefficients when deploying LSTMs for an ED architecture). Although modest, LSTMs also show a better performance for these same features in static mixers for both network frameworks, with average *R*^2^ improvements around 5–11%. This can be related to the simpler and shorter time series studied for static mixers compared to their stirred counterpart. When looking at the more complex data generated for the DSD bins, even though both models tend to struggle to predict some of these dependencies, LSTMs still demonstrate superiority in capturing the variability within specific bins, notably for bins *B*_3_, *B*_5_ and *B*_6_.

In terms of computational requirements during tuning, surprisingly the LSTM and GRU models have similar peak memory usage but GRUs consume significantly more time than LSTMs, particularly in ED models, taking twice as long to tune when dealing with stirred vessels. Only GRUs with an FC architecture for static mixers achieve a lower tuning time, with a slim difference of 3%. Training times show a slightly different conclusion, with shorter training times for GRUs ED in static mixers, but again a general majority of lower training times for LSTM models. Based on the positive points raised for LSTMs in this subsection, we will move forward with analyzing these models in further detail and exploring their performance and robustness.

### Model Generalization: Performance on Training and Validation Data

Before analyzing model performance using LSTM cells on the testing sets, we first compare the performance of both trained networks on the training and validation data sets, depicted in Figures 9 and 10, where the scattered points represent the predicted values for all the features studied. It is observed that most of the points are located at the left bottom corner. This region in the figure corresponds to a range of [0, 0.2], wherein most of the feature values of drop density estimation are likely to occur; meanwhile, 10 bins have been considered as individual features which account for more than 80% of the overall 12 features. Hence, it is natural to see the aggregation in this area. Moreover, an evident deviation is displayed in this corner, not only for training and validation but also for testing predictions (refer to Figure 11). Therefore, relevant discussion on this observation will be included below to avoid redundancy. On the other hand, the predicted values relevant to the training data set align reasonably well with the true data; in contrast, a deviation between predictions and the ground truth slightly develops when looking at the validation set. This conclusion can be supported when looking at the corresponding RMSE and *R*^2^ values for the training and validation sets (see Supporting Information for further detail). The consistently small values of RMSE for both the stirred mixer (0.057 training and 0.0578 validation) and static mixer (0.032 training and 0.046 validation), coupled with the marginal increase in values for validation relative to those for training data set, suggest that the trained LSTM-FC model adeptly captures the underlying patterns in the dispersion dynamics and can generalize this knowledge to new data, indicating robustness against overfitting and ensuring reliable predictions on unseen data. A similar performance can be seen from the RMSE values for the LSTM-ED, which is also supported by the corresponding values of *R*^2^. From the dispersion clouds displayed in Figures 9 and 10, no major differences are apparent between the two architectures when examining the static mixer. On the contrary, the LSTM-ED seems to provide a better-trained state for the stirred vessel, as most of the sequences above 20% deviation are eliminated, especially for the clean and low Bi (<0.1) cases. This observation holds for the validation set, where only a small section is underpredicted beyond a 20% deviation for the encoder-decoder structure, unlike the LSTM-FC, which heavily overpredicts the surfactant-laden case.

### Model Performance: Prediction via Rollout

This section dives into the model rollout predictions on the testing data sets for both mixing systems and LSTM architectures. Similar to what we have shown for the training and validation data sets, Figure 11 presents error dispersion plots for the testing cases, which contain all 12 target scaled features (ND, IA, and 10 bins). This figure offers a broad overview of each model’s performance for all testing cases. However, it falls short when pinpointing the specific features associated with a particular error cloud or region. This association can only be achieved by correlating it with the subsequent figures showcasing the rollout predictions for each feature (Figures 12 and 13).

Starting with the LSTM-FC model for the static mixer in Figure 11b, the dots corresponding to the surfactant-free case of Fine Pre-Mix (colored light yellow) are mostly found above the black solid parity line (*y* = *x*). This implies that the model is inclined to overestimate the dispersion metrics for this particular setup, predicting a larger interfacial area, and more, larger dispersed drops overall. We believe that the overestimation is due to the trained LSTM failing to learn the relationship between the surfactant and the distribution of ND and IA, whereby the absence of the surfactant in the fine pre-mix case naturally leads to markedly smaller ND and IA values due to higher interfacial tension when compared to other surfactant cases.

Another example is the Alt 2 static mixer case shown in Figure 11b, where its predicted values (colored red–orange) are entirely underestimated, signifying that the distinct dispersion performance patterns induced by an alteration in the mixer’s geometry, rather than a change in the chemical nature of the species (e.g., surfactant profile), is not learned properly. In contrast, the predicted values via LSTM-FC for the stirred mixer cases are distributed over both sides of the parity line *y* = *x*, as shown in Figure 11a, implying that there is not a defined bias in the accuracy of the model. Figure 12 offers a detailed perspective on the rollout predictions concerning each feature. In all cases, encompassing both mixers, noticeable variations beyond ±20% are observed at low true data values (data point < 0.3). These discrepancies predominantly align with early time-step predictions for any feature. Specifically, this deviation is most likely due to the sensitivity of DSD to perturbations in the total drop count during the early stages of dispersion formation, especially at the edges of the distribution (i.e., small or large drops). More discussion relevant to this will be presented along with subsequent figures.

As mentioned previously, Figure 12 compares the target and predicted values for each feature via LSTM-FC. The initial observation drawn from this figure indicates that the predicted values for features ND and IA (at early times) exhibit a much better agreement with the targets compared to that seen for the selected bins exemplified herein. This corroborates our earlier assertion regarding the origin of the large deviations associated with low-value true data. Moreover, the trained LSTM-FC correctly captures the hierarchy of the stirred mixer’s cases for all features (see Figure 12a,c,e), particularly at the early time-steps of the predicted sequence. Taking ND as an example, the order, Cl: 8 Hz > Bi = 0.002 > Bi = 1, is then reproduced for time-steps ∼50–200. However, the trained LSTM-FC is unable to predict this ranking further down the time range, as displayed in Figure 12a: the predicted ND for Cl: 8 Hz is lower than that for Bi = 0.002. This can be attributed to the model’s inability to extract the correct physical knowledge from the Cl: 8 Hz case, failing to recognize that this particular case has a higher impeller speed, thus resulting in an extended duration of dispersed drop generation. A similar scenario can be seen for the static mixer (see Figure 12b,d,f), wherein the trained LSTM-FC is capable of recovering the hierarchy at early times, but since the gap between the prior input information and the point where it is needed becomes larger, the model starts to perform poorly to some degree.

The overall performance of the LSTM-ED in terms of the stirred mixer (see Figure 11c) is comparable to that of the LSTM-FC, except for the scattered cloud seen at the initial steps, where most of the predicted values are located within the 20% deviation area from the parity line *y* = *x*. However, obvious discrepancies arise from the fine pre-mix and Da = 1 (colored purple) cases when dealing with the static mixer, whereas the predicted values for other cases remain closer to the parity line *y* = *x* (see Figure 11d). As for the performance on each feature, Figure 13 proves that the capability of the LSTM-ED is similar to that of the LSTM-FC, correctly capturing the case hierarchy at the early times. Furthermore, Figure 13b,d,f clearly shows the deviation highlighted above for the static mixer (the case of fine pre-mix) in terms of each feature. This provides evidence to support the fact that the deviation is mainly caused by the poor performance on the bins prediction. From these figures, we infer that the underestimated *B*_3_ and the overestimated *B*_5_ and *B*_6_ correspond to the dots occur beyond ±20% in Figure 11.

From the discussion presented above, it is clear that the prediction accuracy is high at early times and deteriorates subsequently for both the LSTM-FC and LSTM-ED; however, this occurs for different reasons related to the different architectures. As described in the previous section, LSTM-FC is trained to map an entire output sequence with its input, while LSTM-ED learns to progressively generate the output sequence; this could enable the LSTM-ED to have a superior performance when dealing with sequence generation (i.e., prediction via rollout herein) since the LSTM-FC has not been trained to utilize the previous prediction for the generation of the next sequence. Consequently, the LSTM-ED is likely to perform particularly well given that the dispersion dynamics underlying the data have been learned. For instance, as presented in Figure 13c, the trained LSTM-ED accurately predicts the increase in the case of Cl: 8 Hz as well as the descending trend for Bi = 0.002 and Bi = 1.

Nonetheless, additional observations from Figure 13 indicate that the trained LSTM-ED performs relatively poorly on some features (or cases), e.g., the ND predictions exhibit deviations from their target values for various stirred mixer cases. Similarly, for static mixers (e.g., see *B*_5_ in Figure 13f), the case of fine pre-mix deviates substantially from its target, while the trend for Bi_alt4_ = 0.01 is properly captured. These observations suggest that the trained LSTM-ED must be improved to achieve a better understanding of the features and the mechanisms underlying the data presented in this work; this could be achieved by training on larger data sets and further tuning the hyperparameters. Although simply mapping between input and output sequences makes it comparatively easier to train an LSTM-FC, its performance would be globally inferior to that of a well-trained and finely tuned LSTM Encoder-decoder; this is due to the feature evolution embedded in the sequence in the latter, which is absent in LSTM-FC.

**Figure 14:**
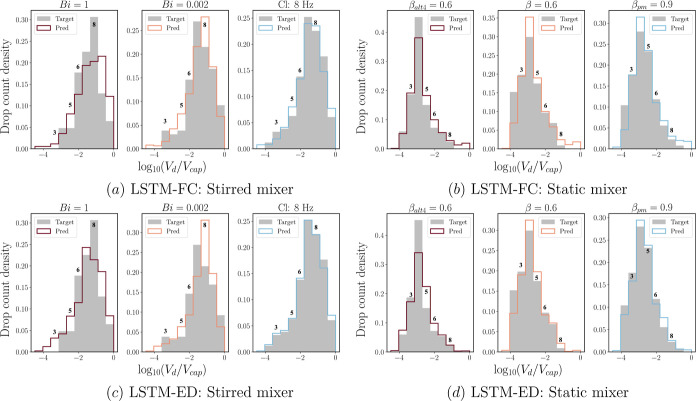
Exemplified histograms presenting the predicted drop size distribution via LSTM-FC (a,b) and LSTM-ED (c,d) for both mixers. In the case of stirred mixer, three test cases are shown for time-step *t* = 280: *Bi* = 1, *Bi* = 0.002, and Cl: 8 Hz, whereas the results of static mixer are demonstrated using test cases, β_alt4_ = 0.6, β = 0.6, and β_pm_ = 0.9 at time-step *t* = 74. The numeric labels in the figure denote the corresponding bins (*B*_3_, *B*_5_, *B*_6_, and *B*_8_) shown in Figures 12 and 13.

Figure 14 presents examples of the predictions of the DSD in the form of histograms containing all the bins involved in the predictions. This figure, along with the rollout predictions relevant to bins presented above, forms an integrated visualization of model performance concerning DSD prediction. For the case of stirred vessels, it can be seen from Figures 12e and 13e that the two models perform reasonably well on the bins of Cl: 8 Hz, which agrees with what is shown in Figure 14a,c that the predicted distribution of Cl: 8 Hz fairly matches its target. Likewise, from the latter figure, *B*_8_ for the case of *Bi* = 1 is underestimated which is consistent with that displayed in the previous plots.

To give an overview of the difference between the targeted and predicted distributions, we computed the Wasserstein distance using the built-in functions from SciPy library in Python, which is a measurement originating from studying optimal transport problems.^63^ Intuitively, if each distribution is treated as piles of soils, this metric quantifies the masses and corresponding distance that must be moved around to turn one distribution into the other; hence, this metric is also known as earth mover’s distance (EMD). It is ubiquitous in the field of statistical analysis to address the dissimilarity quantification between two probability distributions (see a comprehensive review by Panaretos and Zemel^64^).

**Figure 15:**
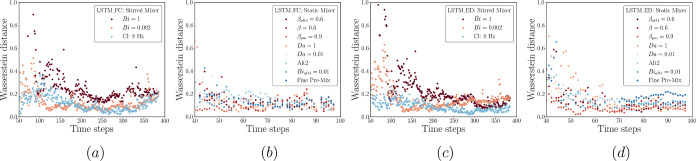
Temporal plots presenting the divergence between targeted and predicted drop size distribution via LSTM-FC (a,b) and LSTM-ED (c,d) for both mixers using the Wasserstein distance.

In general, the EMD can be considered as the distance (or divergence) between two distributions; hence, lower values of this score indicate higher similarity. With this in mind, the information drawn from Figure 15 is that, initially, the predicted DSDs via both LSTM-FC and LSTM-ED tend to significantly deviate from the target; the deviation then slowly diminishes with increasing time-step. This strongly supports our previous hypothesis that the small amount of dispersed drops produced during the early stages of dispersion formation gives rise to a “statistically unstable” distribution that is rather sensitive to small changes in the drop count of each bin. In addition, the statistical instability could be inherently linked to the grid resolution of our simulations, which indicates that nonphysical drops are appearing or disappearing from the domain, causing stochastic irregularity in the data. Recall that as shown in Figure 11, the poor performance related to bins at the early stages of dispersion formation is mirrored by the widely spread dots in the lower left corner of the figure. Nevertheless, with an increase in drop count, the trained models capture the DSD more faithfully with a low level of dissimilarity as reflected by the EMD scores (<0.2) in Figure 15.

### Uncertainty Quantification

In this section, we present the uncertainty quantification of the two trained models, by demonstrating the procedure using two features ND and IA of the test case *Bi* = 0.002 for a stirred mixer and β = 0.6 for a static mixer.

**Figure 16:**
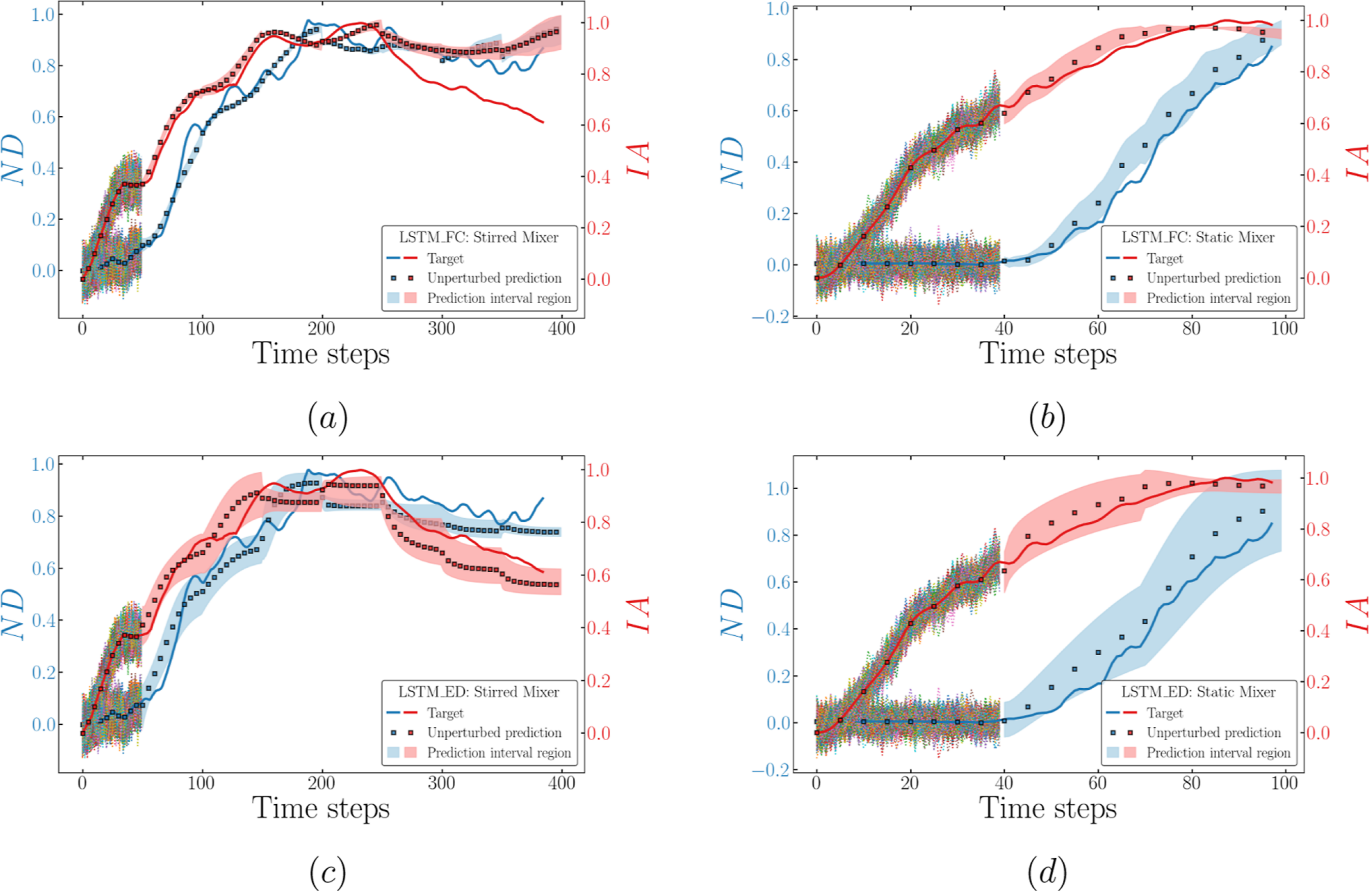
Uncertainty quantification plots, comparing the model target, model predictions from unperturbed input and the ensemble prediction interval region, for LSTM-FC (a,b) and LSTM-ED (c,d). The dots cloud displayed at the early times represents the ensemble of perturbed input sequences.

The results shown in Figure 16 correspond to a comparison among the model target, model prediction from an unperturbed input sequence, and the prediction interval region from a perturbed input ensemble. The first observation from this figure is the different spread sizes of the prediction interval region for the two model architectures. As illustrated previously, perturbation noise is sampled from the distribution , which generates a spread of the input ensemble with corresponding std. dev., σ_s.d._ ≈ 0.04. However, the spread of the prediction ensemble via LSTM-FC is narrower than that of the input ensemble. This suggests a low level of sensitivity of the LSTM-FC to input noise. Meanwhile, an apparent deviation of the target (solid line) from the spread is seen, particularly in the case of the stirred mixers; in comparison, the spread relating to the static mixer captures the target trends for longer periods.

A possible explanation of LSTM-FC’s improved performance for static mixers is that more simulation cases and shorter data lengths are involved, which forms a relatively less challenging learning task. As for the case of stirred mixers, wherever LSTM-FC must deal with longer sequences, either larger training data sets and/or more a sophisticated model is required for a better performance. In contrast, the LSTM-ED presents a wider prediction interval region for both mixers, which mirrors a higher sensitivity to the added noise. Nonetheless, this wider spread correctly captures the target evolution of features relevant to stirred mixers. Such an improvement in performance as well as the higher sensitivity could be attributed to the sequential procedure of the LSTM-ED when generating an output sequence: the model is trained to learn the trend step-by-step; therefore, fluctuations in feature values have a profound effect on the model prediction for the next time-step.

**Figure 17:**
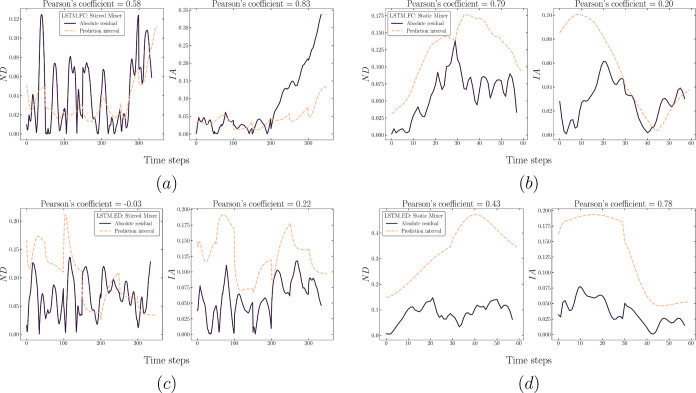
Temporal plots comparing the evolution of spread size and model performance (i.e., absolute residual between model prediction and target at each time-step, ) for both architectures [LSTM-FC in (a,b) and LSTM-ED in (c,d)].

Furthermore, we investigate the correlation between the spread size of the prediction interval region and the model performance along the time axis; here, the model performance is expressed in terms of the divergence of the prediction from its corresponding target at each time-step, which is simply calculated as . Pearson’s correlation coefficient (PCC)^65^ is also computed to compare the strength and direction of the relationship between the prediction and the associated target. As presented in Figure 17, in terms of the PCC (≥0.4), the LSTM-FC-based model performance for the features obtained from both mixers is well captured by the spread size. Similarly, the spread size generated via the LSTM Encoder-decoder in the case of the static mixer presents a fairly strong positive correlation with the model performance indicated by a PCC ≥ 0.4. However, the PCC values relevant to the stirred mixer are relatively low, especially for the feature ND, PCC = −0.03. Nonetheless, related studies have suggested that researchers should not rely on the correlation coefficients for identifying the relation between data sets;^66,67^ instead, it is essential to plot the data for visual inspection.^65,68^ From this perspective, plots shown in Figure 17 provide an insight into the temporal similarity between the spread size and the model performance. It can be seen that, though the spread size does not mirror the exact amplitude of the model performance, it can correctly capture the core trend of the latter, for instance, the exponential increase in IA of the stirred mixer (see Figure 17a) and the subsequent increase following the reduction in IA of static mixer (see Figure 17b). More importantly, we note that the local extrema of the model performance and spread size align (see Figure 17c). Thus, the extrema of the spread size signals the deviation of model predictions from the target. This is beneficial when performing sequential prediction since the spread size could help to identify the point where the accuracy of the model prediction begins to deteriorate.

From the above, we suggest that the spread size of the prediction interval region provides an appropriate indicator of model performance and reliability along the sequence generation. This is particularly valid in the case where the trained model is deployed to predict mixing systems that had not been included in the training data set and for which no access to the model target, or “truth”, is possible.

## Conclusions

The current study has demonstrated the application of the RNN to predict the temporal behavior of key dispersion performance metrics for complex multiphase mixing systems. Specifically, two network architectures, fully connected (FC) and encoder–decoder, with two different RNN cells, LSTM and GRU, have been finely trained and deployed to predict interfacial area growth, drop count, and their size distribution for future time-steps. The RNN models developed herein have been trained with data obtained from high-fidelity, three-dimensional, CFD simulations of two mixing systems, carried out for stirred^28,29^ and static^30,31^ mixers. In particular, the RNN model has been assigned the task of learning and capturing the influence of varying physicochemical properties, operational conditions, and mixer geometrical characteristics on dispersion performance. Concerning the network architecture, the RNN-FC has been taught to map a single long output sequence from the input sequence alone, while the RNN-ED has learned to progressively generate future output sequences based on its previous predictions. The findings presented herein showed very similar performances between GRU and LSTM units in terms of accuracy and computational resource consumption, although a slight edge was observed for LSTMs, as previously discussed. This advantage can be attributed to the architectural distinction where LSTMs handle both cell and hidden states, unlike GRUs, which solely manage hidden states. This implies that LSTM units are capable of better handling long-term dependencies and intricate data evolution patterns.

Focusing our attention on LSTM-based networks, we have presented the model performance in terms of the prediction of each dispersion metric obtained for stirred and static mixers. For both model architectures, the prediction accuracy is high at early times and subsequently deteriorates. We have reasoned this to the nature of the rollout procedure, wherein the entire dispersion metrics evolution is extrapolated based solely on their initial behavior during the early stages of the process. In addition, we have demonstrated that it is easier to train an LSTM-FC due to its simplicity, although inferior model performance has been observed because the feature evolution embedded in the sequence is absent. By contrast, the trained LSTM-ED is capable of recovering the dispersion dynamics that is incorrectly predicted when the trained LSTM-FC is applied. However, we have suggested that the trained LSTM-ED in the current work must be improved since nontrivial deviation on some features is seen. In particular, we present different methods to visualize the model performance relevant to the drop size distribution. These methods include the temporal drop counts in each bin representing a specific drop size range and the histogram of all bins at one time-step. Additionally, Wasserstein distances have been computed to track the similarity between the predicted and targeted distributions. With this score, we have shown that the evident discrepancies occur at the early times, where the corresponding DSD is sensitive to the perturbation in the total drop count due to its small value initially; hence, the deviation gradually diminishes with the increase in drop numbers. Lastly, we have proposed an ensemble-based procedure to quantify the model uncertainty, where we have further investigated the correlation between the spread size of the prediction interval region and the model performance during prediction via rollout. We have illustrated the high sensitivity of the LSTM-ED when concerning the added noise drawn from the same Gaussian distribution as LSTM-FC; meanwhile, the prediction interval via the former verifies its robustness by correctly capturing the true data. Moreover, throughout the correlation investigation, we suggest that the spread size could serve as an appropriate indicator of the evolution of model reliability through the rollout.

Throughout this work, we have delineated a baseline procedure for data preprocessing, reconditioning, and RNN deployment in the field of complex multiphase mixing. More importantly, we have implemented a system-agnostic framework capable of seamlessly handling any time-series data with similar structures from other applications, regardless of their origin (i.e., numerical data or experimental measurements), and performing analogous temporal evolution predictions of key metrics. Thus, this study could significantly benefit the chemical engineering academic community and industrial practitioners by introducing a cost-effective yet robust alternative to computationally demanding or high-fidelity CFD modeling frameworks, such as those presented in our previous works (i.e., interface-tracking methods), intended to provide an accurate representation of complex interfacial metrics as the ones studied herein. As discussed in this work, the RNNs deployed can certainly outperform, in terms of time consumption for fine-tuning, training, and deploying high-fidelity interface-resolving CFD methods, while still providing accurate predictions for these complex metrics, as examined previously in Table 4.

A spectrum of additional hyperparameters not considered in this work could also affect the model performance, such as the number of LSTM layers, different learning rate schedulers, or dynamic teacher forcing (*d*.*t*.*f*.). Future work will explore optimal configurations for RNNs and implementing variations in the model’s architecture. Additionally, investigation regarding uncertainty quantification can be extended by considering various types of uncertainties^69,70^ to test the model’s capability of filtering noises from different sources.

## Supporting Information Available

Comparison of performance of LSTM-FC, LSTM-ED, GRU-FC, and GRU-ED and counterpart plots of Figures 9–15 surrounding RNN-GRU (PDF)
