# Supplementary material for: Liquid–Liquid Dispersion Performance Prediction and Uncertainty Quantification Using Recurrent Neural Networks
Source: Ind Eng Chem Res. 2024 Apr 22;63(17):7853–75. doi: 10.1021/acs.iecr.4c00014 (PMC11066846; doi:10.1021/acs.iecr.4c00014)
Supplement: Supplementary file 1 — ie4c00014_si_001.pdf [file ie4c00014_si_001.pdf]

Supporting Information

Liquid-liquid dispersion performance prediction and uncertainty quantification using recurrent neural networks

Fuyue Liang,*,† Juan P. Valdes,† Sibo Cheng,‡ Lyes Kahouadji,† Seungwon Shin,¶ Jalel Chergui,§ Damir Juric,§,‖ Rossella Arcucci,⊥ and Omar K. Matar†

†*Department of Chemical Engineering, Imperial College London, London, SW7 2AZ, UK*

‡*Data Science Institute, Imperial College London, London, SW7 2AZ, UK*

¶*Department of Mechanical and System Design Engineering, Hongik University, Seoul, 04066, Republic of Korea*

§*Université Paris Saclay, Centre National de la Recherche Scientifique (CNRS), Laboratoire Interdisciplinaire des Sciences du Numérique (LISN), Orsay, 91400, France*

‖*Department of Applied Mathematics and Theoretical Physics, University of Cambridge, Cambridge, CB3 0WA, UK*

⊥*Department of Earth Science & Engineering, Imperial College London, London, SW7 2AZ, UK*

E-mail: fuyue.liang18@imperial.ac.uk

In this Supporting Information file, we first provide the comparative performance, in terms of model accuracy and computational requirement, for the four types of models we discussed in the manuscripts (i.e., LSTM_FC, LSTM_ED, GRU_FC, GRU_ED) relevant to the training, validation and testing datasets; Table S1 and Table S2 present the model accuracy (RMSE and R^2) and computational performance metrics (time and memory requirements) for stirred and static mixers, respectively. In addition, model generalisation and performance on the testing datasets for the cases where GRU units are implemented are presented throughout Figure S1-Figure S7 as counterparts of Figure 9 - Figure 15 in the main manuscript.

Table S1: Model accuracy and computational performance metrics for the stirred mixer, divided by network architecture (FC/ED) and subdivided by RNN unit type (LSTM/GRU). R^2 and RMSE values presented in brackets correspond to the training, validation and testing sets, respectively.

**Metrics**	**Stirred mixer**			
	**LSTM_FC**	**LSTM_ED**	**GRU_FC**	**GRU_ED**
**Overall RMSE**	(0.057, 0.058, 0.063)	(0.050, 0.050, 0.066)	(0.063, 0.077, 0.065)	(0.063, 0.047, 0.074)
**Overall** R^2	(0.952, 0.951, 0.943)	(0.963, 0.963, 0.938)	(0.941, 0.913, 0.939)	(0.942, 0.967, 0.921)
R^2 (ND)	(0.854, 0.847, 0.951)	(0.889, 0.863, 0.915)	(0.743, 0.578, 0.923)	(0.831, 0.891, 0.820)
R^2 (IA)	(0.383, 0.774, 0.877)	(0.856, 0.783, 0.877)	(0.499, 0.178, 0.830)	(0.591, 0.914, 0.711)
R^2 (B_3)	(0.382, -0.419, 0.570)	(0.423, 0.083, 0.575)	(0.202, -0.577, 0.552)	(0.375, -0.065, 0.613)
R^2 (B_5)	(-0.227, -1.381, -0.051)	(0.005, -0.902, -0.042)	(-0.851, -3.840, -0.366)	(0.172, -2.830, 0.591)
R^2 (B_6)	(-0.601, -2.582, 0.600)	(0.192, -3.331, 0.416)	(-0.347, -8.812, 0.567)	(0.130, -1.195, 0.579)
R^2 (B_8)	(0.655, 0.010, 0.691)	(0.409, 0.207, 0.597)	(0.317, -3.182, 0.780)	(0.522, -0.387, 0.805)
**Tuning time (mins)**	4915	7310	5726	12712
**Tuning peak memory (MB)**	124.33	124.68	124.27	124.55
**Training time (mins)**	59	448	272	618
**Training peak memory (MB)**	13.44	13.44	13.44	13.44

Table S2: Model accuracy and computational performance metrics for the stirred mixer, divided by network architecture (FC/ED) and subdivided by RNN unit type (LSTM/GRU). R^2 and RMSE values presented in brackets correspond to the training, validation and testing sets, respectively.

**Metrics**	**Static mixer**			
	**LSTM_FC**	**LSTM_ED**	**GRU_FC**	**GRU_ED**
**Overall RMSE**	(0.032, 0.046, 0.037)	(0.042, 0.049, 0.046)	(0.032, 0.045, 0.039)	(0.035, 0.051, 0.048)
**Overall R^2**	(0.985, 0.967, 0.979)	(0.974, 0.964, 0.969)	(0.986, 0.969, 0.977)	(0.982, 0.961, 0.966)
R^2 (ND)	(0.974, 0.874, 0.976)	(0.920, 0.884, 0.946)	(0.972, 0.878, 0.965)	(0.959, 0.855, 0.942)
R^2 (IA)	(0.909, 0.918, 0.995)	(0.898, 0.932, 0.993)	(0.933, 0.943, 0.994)	(0.895, 0.818, 0.991)
R^2 (B_3)	(0.558, -0.112, 0.854)	(0.529, 0.176, 0.844)	(0.621, -0.079, 0.848)	(0.519, 0.155, 0.823)
R^2 (B_5)	(0.861, 0.544, 0.931)	(0.796, 0.460, 0.896)	(0.813, 0.477, 0.915)	(0.808, 0.254, 0.848)
R^2 (B_6)	(0.485, -1.916, 0.531)	(0.347,-2.483, 0.665)	(0.570, -1.390, 0.573)	(0.498, -11.445, -0.784)
R^2 (B_8)	(0.751, 0.457, 0.987)	(0.539, -0.041, 0.981)	(0.786, 0.480, 0.986)	(0.695, -0.345, 0.941)
**Tuning time (mins)**	614.900	1366.947	595.482	1498.787
**Tuning peak memory (MB)**	157.398	186.001	161.493	187.716
**Training time (mins)**	7.234	162.421	14.737	117.984
**Training peak memory (MB)**	11.158	11.175	11.157	11.176

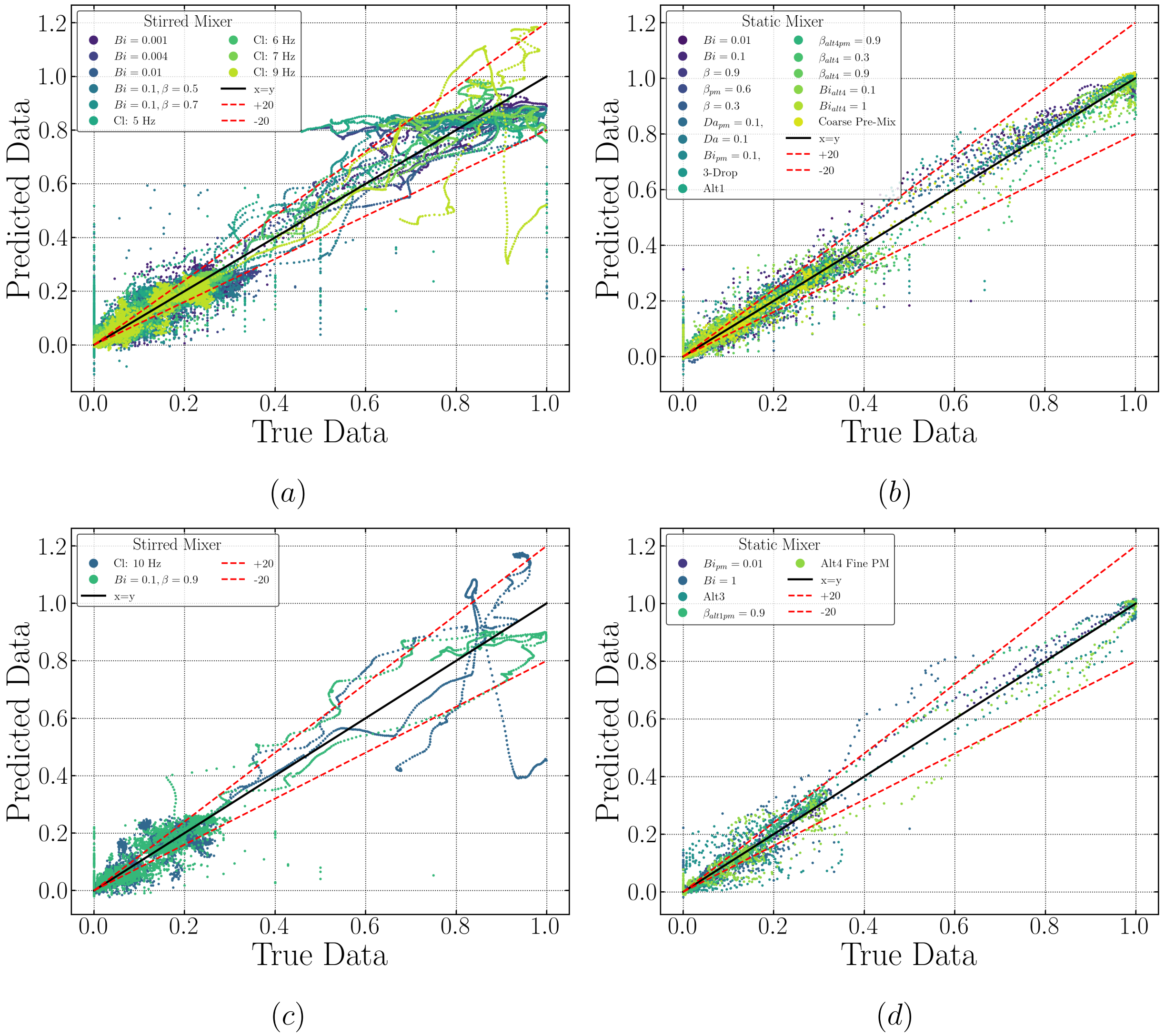

Figure S1: GRU-FC predicted vs. true data error dispersion plots for all 12 features considered in this study. A $\pm$ 20 % deviation area is included. Sub-figures to the left ((*a*), (*c*)) showcase training and validation data for stirred mixers, while those to the right ((*b*), (*d*)) illustrate training and validation data for static mixers, respectively.

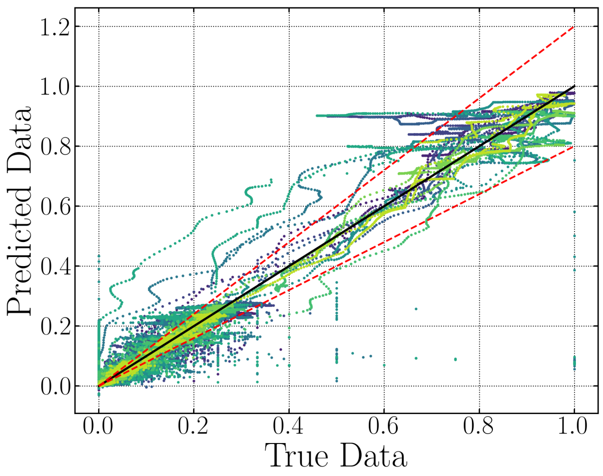

(a)

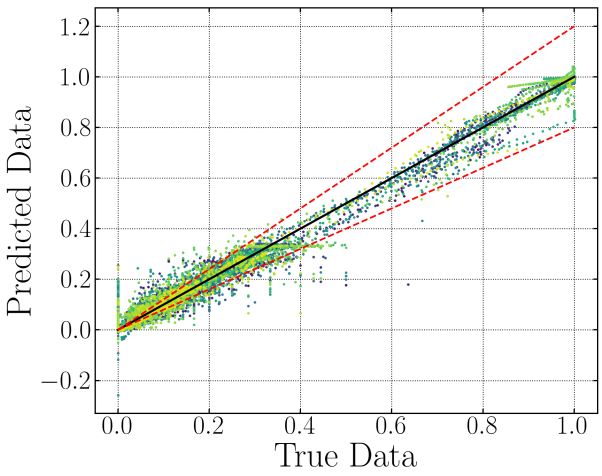

(b)

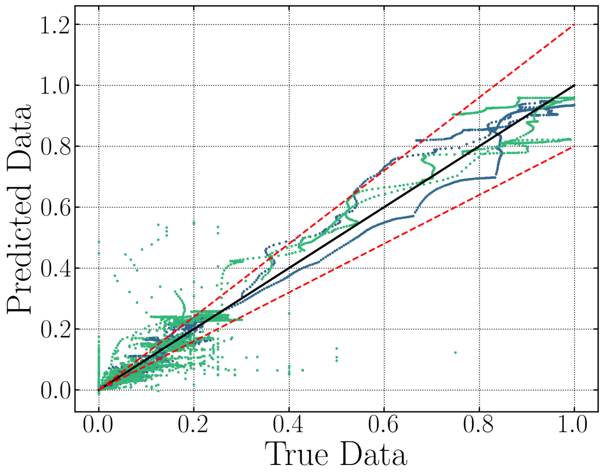

(c)

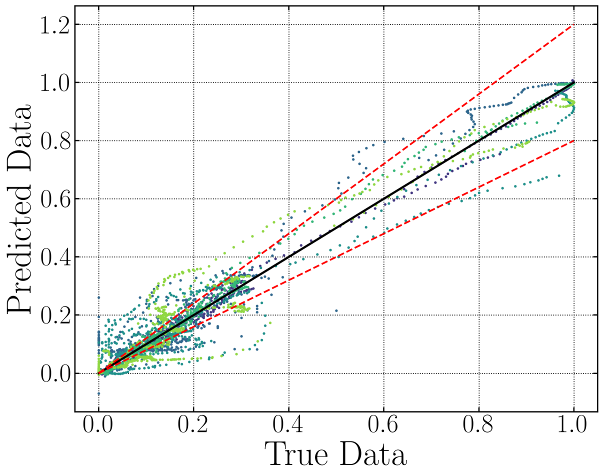

(d)

Figure S2: GRU-ED predicted vs. true data error dispersion plots for all 12 features considered in this study. A $\pm 20\%$ deviation area is included. Sub-figures to the left ((a), (c)) showcase training and validation data for stirred mixers, while those to the right ((b), (d)) illustrate training and validation data for static mixers, respectively. Plot legends are shared with **??**, and thus not included here to avoid redundancy.

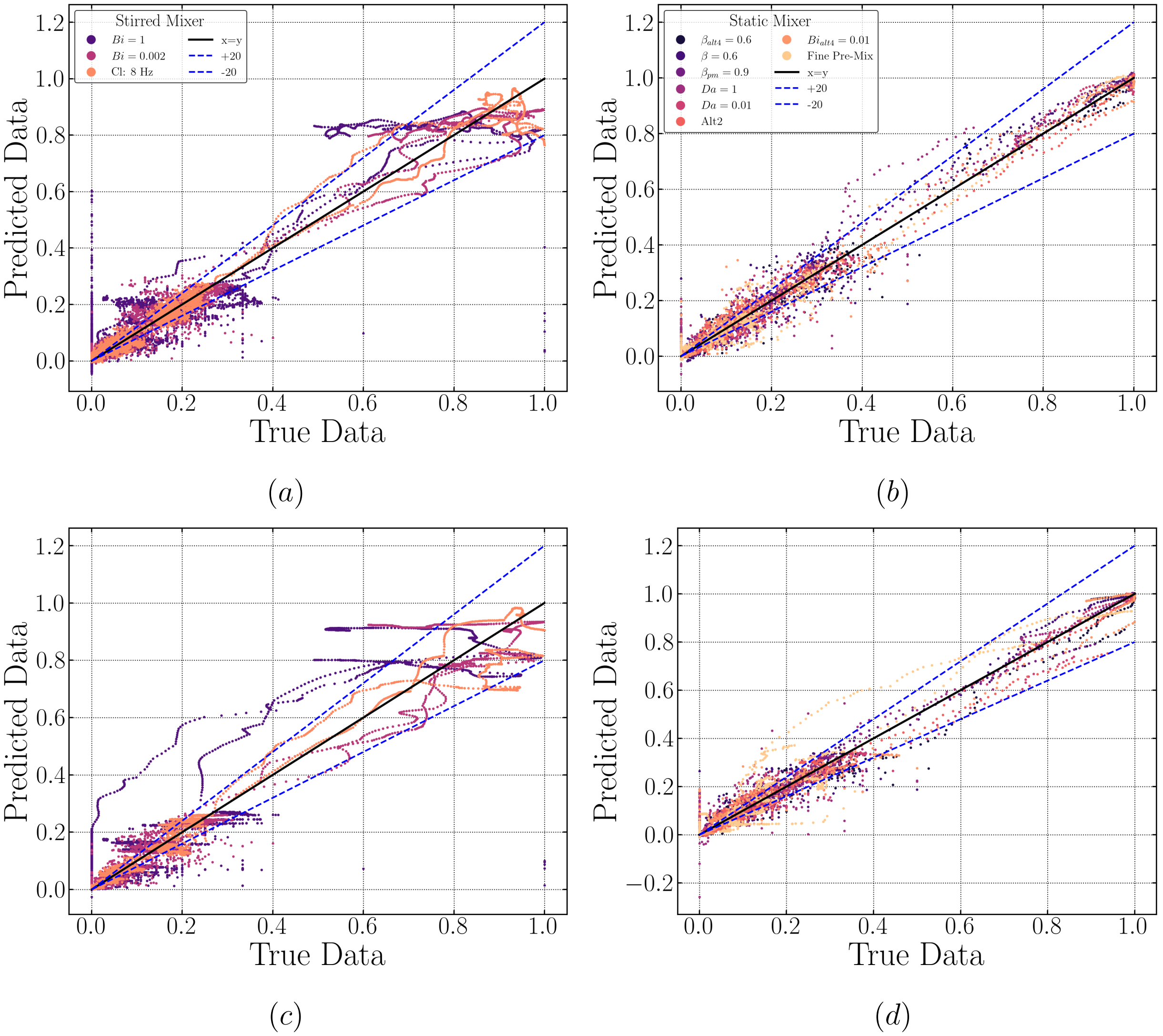

Figure S3: Predicted vs true data error dispersion plots for testing datasets, with a ±20% deviation region included. Sub-figures (a) and (c) showcase rollout prediction data dispersion for the stirred mixer via FC and ED architecture, while subfigures (b) and (d) illustrate rollout prediction data for the static mixer via FC and ED, respectively.

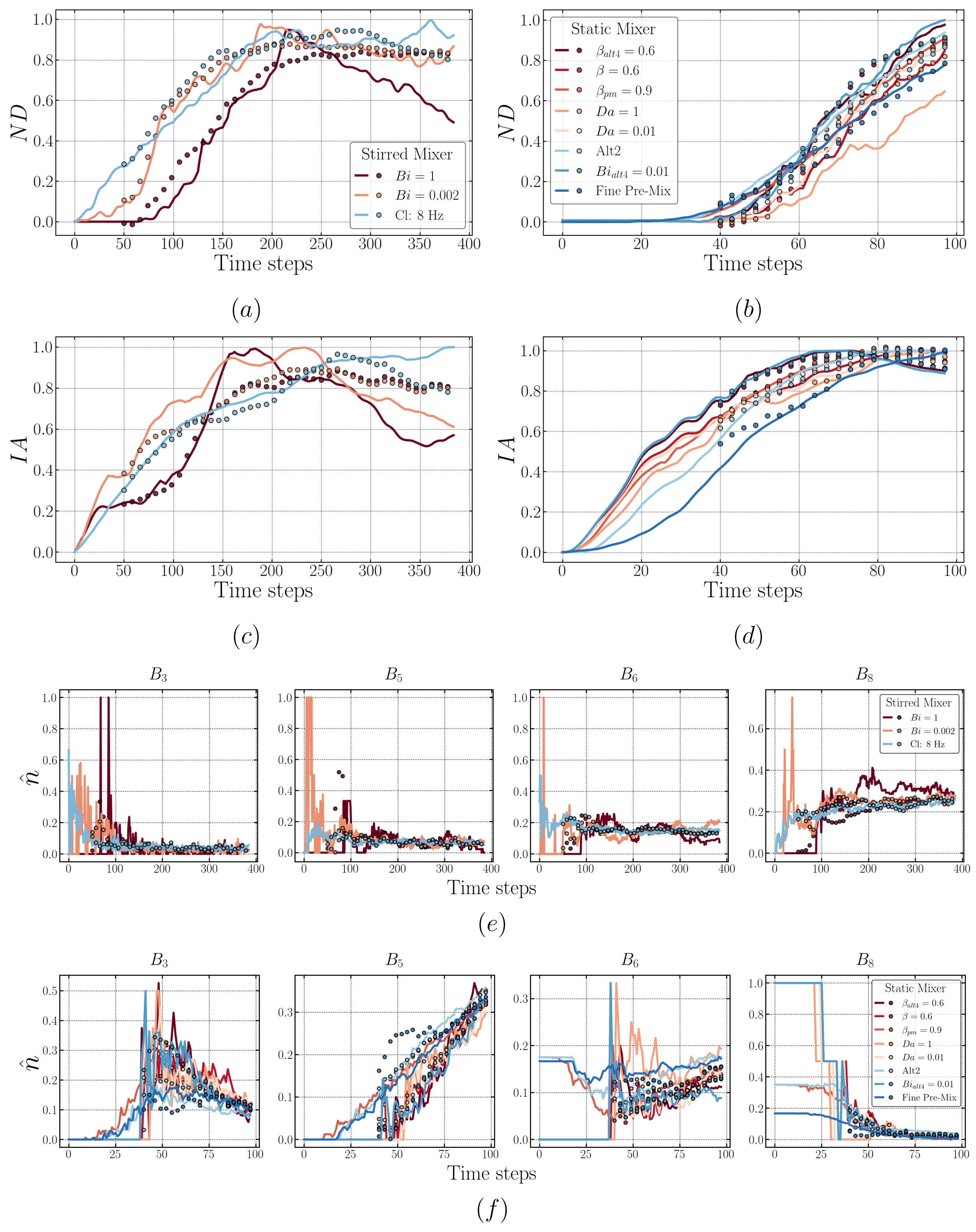

Figure S4: Plots comparing the model target sequences (lines) and predicted sequences via rollout procedure (dots) of GRU-FC for both mixers (left plots (a), (c) correspond to the stirred mixer, (b), (d) correspond to the static mixer, with features ND and IA in the top and bottom, respectively). The results of predicted drop size distribution are exemplified using B_3, B_5, B_6 and B_8 for both mixers $((e), (f))$.

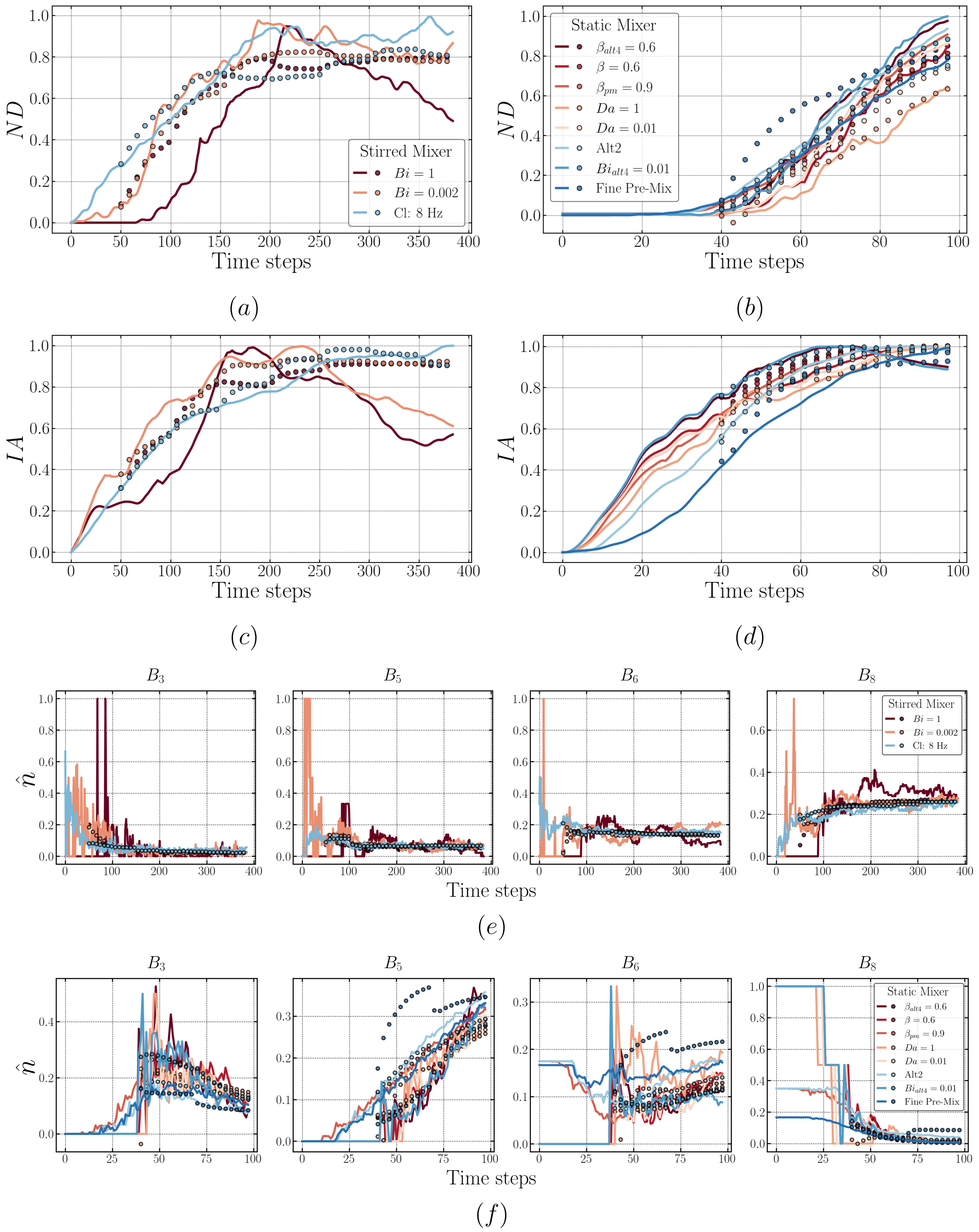

Figure S5: Plots comparing the model target sequences (lines) and predicted sequences via rollout procedure (dots) of GRU-ED for both mixers (left plots (a), (c) correspond to the stirred mixer, (b), (d) correspond to the static mixer, with features ND and IA in the top and bottom, respectively). The results of predicted drop size distribution are exemplified using B_3, B_5, B_6 and B_8 for both mixers $((e), (f))$.

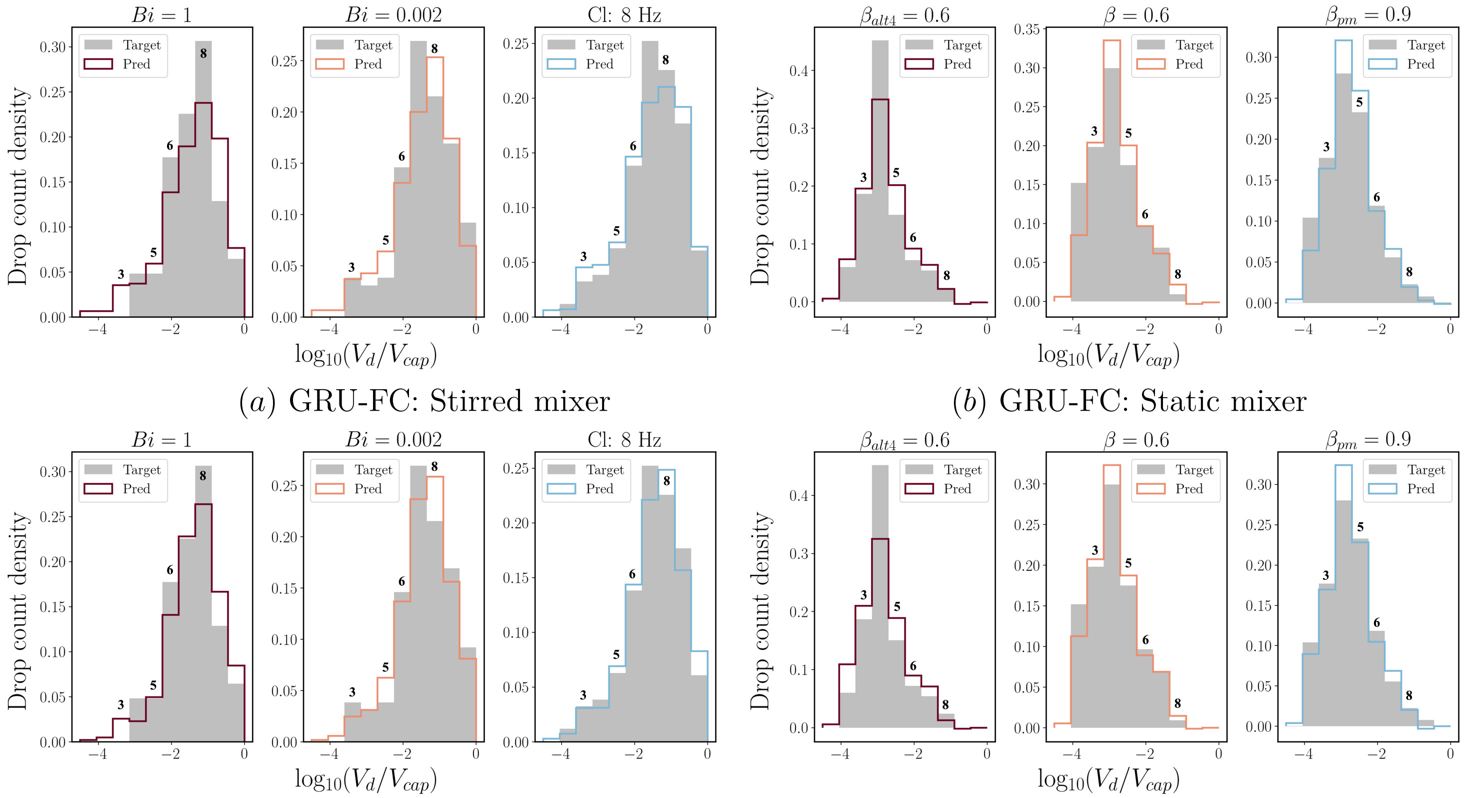

(a) GRU-FC: Stirred mixer (b) GRU-FC: Static mixer

(c) GRU-ED: Stirred mixer (d) GRU-ED: Static mixer

Figure S6: Exemplified histograms presenting the predicted drop size distribution via GRU-FC $((a),(b))$ and GRU-ED $((c),(d))$ for both mixers. In the case of stirred mixer, three test cases are shown for time-step $t = 280$: $Bi = 1, Bi = 0.002$ and Cl: 8 Hz, whereas the results of static mixer are demonstrated using test cases, $\beta_{\text{alt4}} = 0.6, \beta = 0.6$ and $\beta_{\text{pm}} = 0.9$ at time-step $t = 74$. The numeric labels in the figure denote the corresponding bins (B_3, B_5, B_6 and B_8) shown in Figure S4 and Figure S5.

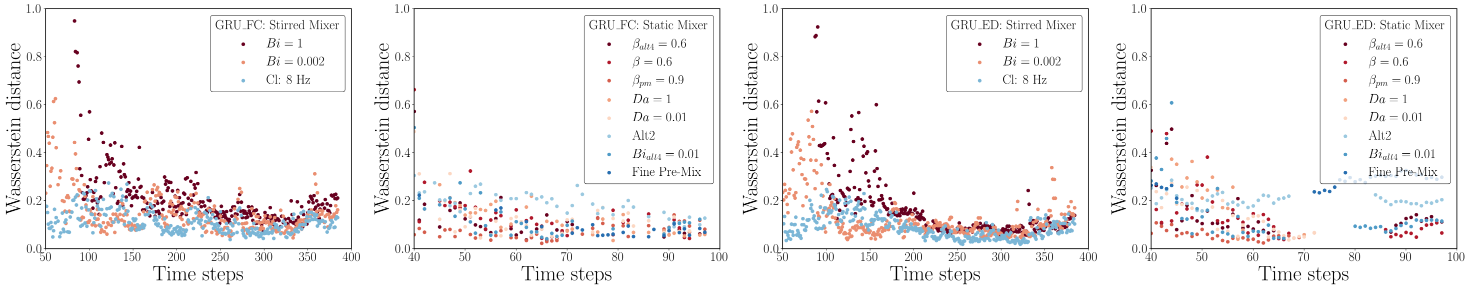

(a) (b) (c) (d)

Figure S7: Temporal plots presenting the divergence between targeted and predicted drop size distribution via GRU-FC $((a),(b))$ and GRU-ED $((c),(d))$ for both mixers, using metrics, Wasserstein distance.
